# ﻿Morphological and phylogenetic analyses reveal five new species of Porotheleaceae (Agaricales, Basidiomycota) from China

**DOI:** 10.3897/mycokeys.105.118826

**Published:** 2024-04-25

**Authors:** Qin Na, Hui Zeng, Yaping Hu, Hui Ding, Binrong Ke, Zhiheng Zeng, Changjing Liu, Xianhao Cheng, Yupeng Ge

**Affiliations:** 1 Institute of Mycological Science and Technology, School of Agriculture, Ludong University, Yantai 264025, China Ludong University Yantai China; 2 Institute of Edible Fungi, Fujian Academy of Agricultural Sciences; National and Local Joint Engineering Research Center for Breeding & Cultivation of Features Edible Fungi, Fuzhou 350014, China Fujian Academy of Agricultural Sciences Fuzhou China; 3 Nanjing Institute of Environmental Sciences, Ministry of Ecology and Environment, State Environmental Protection Scientific Observation and Research Station for Ecological Environment of Wuyi Mountains, Nanjing 210042, China Nanjing Institute of Environmental Sciences Nanjing China; 4 College of Criminal Science and Technology, Nanjing Police University, Nanjing 210042, China Nanjing Police University Nanjing China

**Keywords:** cyphelloid polypores, new taxon, *Porotheleum*, systematics, white-spored omphalinoid fungi

## Abstract

The first occurrence of *Marasmiellomycena* and *Pulverulina* in the Chinese mycobiota are reported, *M.tomentosa* and *P.flavoalba*, two new species and *M.albodescendens*, a new combination, revealed by phylogenetic analyses and morphological study. These newly-recorded genera, *Marasmiellomycena*, which can be distinguished by their agaricoid basidiomata, dark-coloured stipe, sarcodimitic tramal structure, stipitipellis with yellow to yellowish-brown pigments and yellow-pigmented thick-walled caulocystidia and *Pulverulina*, which differs from other genera of Porotheleaceae by its pruinose stipe, decurrent lamellae, inamyloid basidiospores and absence of hymenial cystidia. We also formally describe three other new species of Porotheleaceae collected from Chinese temperate to subtropical zones of Fujian and Zhejiang Provinces: *Clitocybulafuscostriata*, *Gerronemabrunneosquamulosum* and *Leucoinocybesubglobispora*. Furthermore, we include the results of a phylogenetic analysis of Porotheleaceae, based on a multi-locus (ITS, nrLSU and *rpb2*) dataset. According to this analysis, *Chrysomycena*, *Clitocybula*, *Delicatula*, *Hydropodia*, *Hydropus*, *Leucoinocybe*, *Marasmiellomycena*, *Megacollybia*, *Pulverulina*, *Trogia* and *Vizzinia* are monophyletic. However, *Gerronema* is identified as polyphyletic and, additionally, *Porotheleum* does not form a monophyletic group either because *Porotheleumparvulum* and *Porotheleumalbidum* are “unassigned” in phylogenetic analysis. The results of our phylogenetic analyses, coupled with morphological observations, confirm recognition of these new taxa. Morphological descriptions, photographs, line drawings and comparisons with closely-related taxa are presented for the new species. A key to the 22 species belonging to nine genera of Porotheleaceae in China is also provided.

## ﻿Introduction

The family Porotheleaceae (order Agaricales), formally proposed by Murrill (1916), comprises saprotrophic, mainly wood-decaying fungi that are primarily agarics, but also include cyphelloid fungi. The type genus, *Porotheleum* Fr., is distinguished by fruiting in clusters of small cup-shaped to tubular cream cyphelloid basidiomes, whereas other genera are typically agaricoid (Vizzini et al. 2022). Previous taxonomic studies have included 15 genera in Porotheleaceae: *Chrysomycena* Vizzini, Picillo, Perrone & Dovana, *Clitocybula* (Singer) Singer ex Métrod, *Delicatula* Fayod, *Gerronema* Singer, *Hydropodia* Vizzini & Consiglio, *Hydropus* Kühner ex Singer, *Leucoinocybe* Singer ex Antonín, Borovička, Holec & Kolařík, *Lignomphalia* Antonín, Borovička, Holec & Kolařík, *Marasmiellomycena* De la Peña-Lastra, Mateos, Kolařík, Ševčíková & Antonín, *Megacollybia* Kotl. & Pouzar, *Porotheleum*, *Pulverulina* Matheny & K.W. Hughes, *Pseudohydropus* Vizzini & Consiglio, *Trogia* Fr. and *Vizzinia* Ševčíková & Kolařík (Antonín et al. 2019; Vizzini et al. 2019, 2022; Matheny et al. 2020; Senanayake et al. 2023). Most taxa, except for *Porotheleum*, are well characterised, based on the following features: a saprophytic habit; omphalinoid, collybioid, to clitocyboid basidiomata; partly to entirely pigmented pileus; adnexed, subdecurrent, to decurrent lamellae; smooth, thin-walled basidiospores; and the frequent presence of sarcodimitic tramal tissues (Singer 1951, 1982; Redhead 1987; Norvell et al. 1994; Hughes et al. 2007; Kumar and Manimohan 2009; Yang et al. 2012; Vizzini et al. 2019; Consiglio et al. 2022; Senanayake et al. 2023). Species of Porotheleaceae are widespread in subtropical to tropical regions and tend to be lower diversity in temperate zones (Singer 1951, 1970; Norvell et al. 1994; Antonín and Noordeloos 2004; Hughes et al. 2007; Antonín et al. 2019; Vizzini et al. 2019; Consiglio et al. 2022; Na et al. 2022a; Senanayake et al. 2023). Six new genera have recently been recognised: *Chrysomycena*, *Hydropodia*, *Marasmiellomycena*, *Pulverulina*, *Pseudohydropus* and *Vizzinia* (Vizzini et al. 2019; Matheny et al. 2020; Consiglio et al. 2022; Senanayake et al. 2023). These newly-described genera have been found in diverse regions, predominantly in Europe and North America, with some findings in Oceania, Africa and Asia, but the distribution reflects the broad yet unequal exploration of this family’s species, only one species is from Japan, in Asia and none from China (Cooper 2014; Vizzini et al. 2019; Villarreal et al. 2021; Consiglio et al. 2022; Kasuya et al. 2023; Senanayake et al. 2023). While Index Fungorum (http://www.indexfungorum.org/Names/Names.asp 2023.3.16) records 670 Porotheleaceae species, only seven species are documented from China, indicating a disparity in mycological research within the region (Liu 1995; Yang et al. 2012; Liu et al. 2019; Na et al. 2022a).

A comprehensive phylogenetic analysis of Porotheleaceae has not been performed because few sequences are available. Prior to 2012, the family was informally cited in literature as the ‘hydropoid’ clade within the ‘marasmioid’ clade (Moncalvo et al. 2002; Matheny et al. 2006; Antonín et al. 2019). Many authors have since suggested that members of the hydropoid clade should be placed in the phylogenetically defined Porotheleaceae clade (Henrici 2012; Redhead 2013; Cooper 2016; Vizzini et al. 2019, 2022; Kalichman et al. 2020; Matheny et al. 2020; Senanayake et al. 2023). According to a study based on the large subunit of nuclear ribosomal DNA (nrLSU) sequences (Moncalvo et al. 2002), eight species in five genera (*Clitocybula*, *Gerronema*, *Hydropus*, *Megacollybia* and *Porotheleum*) constitute this hydropoid (/hydropoid) clade. The results of that study also support the placement of *Megacollybia* and *Clitocybula* as close relatives of *Hydropus*. Moncalvo et al. (2002) also proposed that *Gerronemasensu*Singer (1986) was polyphyletic (Lutzoni 1997; Moncalvo et al. 2000), whereas this genus as delineated by Norvell et al. (1994) was monophyletic. However, the type species of *Gerronema* was not included in the molecular phylogeny of Moncalvo et al. (2002). The delimitation of *Gerronema* by Norvell et al. (1994) was based solely on morphology in comparison to an epitype, with emphasis on the presence of sarcodimitic tissue. The hydropoid clade configuration defined by Moncalvo et al. (2002) based on ribosomal LSU is also presented in Bodensteiner et al. (2004). In a multigenic analysis (18S, 5.8S, 25S, *rpb1* and *rpb2*) performed by Matheny et al. (2006), the hydropoid clade included *Clitocybulaatrialba* (Murrill) Singer [currently *Gerronemaatrialbum* (Murrill) Borovička & Kolařík], *Clitocybulaoculus* (Peck) Singer, *Henningsomycescandidus* (Pers.) Kuntze, *Hydnopolyporusfimbriatus* (Cooke) DA Reid (currently *Irpexrosettiformis* C.C. Chen & Sheng H. Wu), *Hydropusmarginellus* (Pers.) Singer, Hydropuscf.scabripes (Murrill) Singer [currently *Mycopanscabripes* (Murrill) Redhead, Moncalvo & Vilgalys], *Megacollybiaplatyphylla* (Pers.) Kotl. & Pouzar and several species formerly placed in *Mycena* (Pers.) Roussel [i.e. *Mycenaauricoma* Har. Takah. (currently *Leucoinocybeauricoma* (Har. Takah.) Matheny), *Mycenaamabilissima* (Peck) Sacc. (currently *Atheniellaamabilissima* (Peck) Redhead, Moncalvo, Vilgalys, Desjardin & B.A. Perry) and *Mycenaaurantiidisca* (Murrill) Murrill (currently *Atheniellaaurantiidisca* (Murrill) Redhead, Moncalvo, Vilgalys, Desjardin & B.A. Perry)]. Henrici (2012) combined *Megacollybia*, *Clitocybula* and *Hydropus*, along with other genera, into the family Porotheleaceae, comprising a total of 19 genera. Redhead (2012, 2013) expanded the ‘hydropoid’ clade by introducing *Atheniella* Redhead, Moncalvo, Vilgalys, Desjardin & B.A. Perry and established the genus *Phloeomana* Redhead within the family Porotheleaceae. Cooper (2016) also believes that *Porotheleum* should belong to the Porotheleaceae family, despite the possibility of misidentification in the sequenced material of *Porotheleumfimbriatum* (generic type). Finally, Antonín et al. (2019) introduced the new genera *Leucoinocybe* and *Lignomphalia*, which were separated from *Clitocybula*. However, it should be noted that Singer (1943) originally proposed *Leucoinocybe* as a provisional name, rendering the use by Antonín et al. (2019) as a validation rather than the establishment of a completely new genus. In an analysis by Vizzini et al. (2019), Porotheleaceae was statistically well supported (MLB = 100%) when only *Hydropus*, *Clitocybula*, *Leucoinocybe*, *Megacollybia*, *Porotheleum*, *Trogia* and some species of *Gerronema* were included. In addition, *Chrysomycena* formed a distinct monophyletic lineage corresponding to a separate genus, sister to a clade formed by *Megacollybia*, *Trogia* and some species of *Gerronema* (Vizzini et al. 2019). Matheny et al. (2020) performed a phylogenetic analysis of a combined ITS–28S dataset of 73 taxa and found that *Delicatula* and *Pulverulina* (representing a new genus) are members of Porotheleaceae*sensu*Vizzini et al. (2019); this was in agreement with the concept of Porotheleaceae*s.l.* of Kalichman et al. (2020), which comprises Porotheleaceae*sensu*Vizzini et al. (2019), *Actiniceps* Berk. & Broome, *Atheniella*, *Calyptella* Quél., Chaetotyphula Corner, *Hemimycena* Singer, *Lignomphalia*, *Phloeomana* and *Scytinotus* P. Karst. Vizzini et al. (2022) considered the family Porotheleaceae to be equivalent to Porotheleaceae*sensu*Vizzini et al. (2019) and included the other taxa in Porotheleaceae*s.l.*Kalichman et al. (2020) in Cyphellaceae Burnett, a sister family to Porotheleaceae. Senanayake et al. (2023) agree with the concept and composition of Porotheleaceae as defined by Vizzini et al. (2019, 2022) and proposed two new genera of the family, *Marasmiellomycena* and *Vizzinia*. Finally, *Hydropussubalpinus* (Höhn.) Singer, which was not aggregated into clade *Hydropus* with high statistical support, was recently treated as *Hydropodiasubalpina* (Höhn.) Vizzini, Consiglio & M. Marchetti by Consiglio et al. (2022). In the same study, *Pseudohydropus* Vizzini & Consiglio was established, with *Pseudohydropusfloccipes* (Fr.) Vizzini & Consiglio designated as the type species, comprising a total of four species.

Seventeen species belonging to seven genera of Porotheleaceae, namely, one species of *Clitocybula* (Singer) Singer ex Métrod, one species of *Delicatula*, seven species of *Gerronema*, four species of *Hydropus*, one species of *Leucoinocybe*, two species of *Megacollybia* and one species of *Trogia*, have been recognised in China as of 2023 (Liu 1995; Dai et al. 2010; Yang et al. 2012; Liu et al. 2019; Wang et al. 2021; Na et al. 2022a). Progress has recently been made in clarifying the status of mycenoid and omphalinoid fungi in China, including the discovery of four new taxa from Anhui, Fujian and Zhejiang Provinces: *Gerronemabaishanzuense* Q. Na, H. Zeng & Y.P. Ge; *G.microcarpum* Q. Na, H. Zeng & Y.P. Ge; *G.zhujian* Q. Na, H. Zeng & Y.P. Ge; and *Leucoinocybelishuiensis* Q. Na, H. Zeng & Y.P. Ge (Na et al. 2021, 2022a). As part of our ongoing research on omphalinoid fungi, we uncovered the first occurrence of two newly-recorded genera, *Marasmiellomycena* and *Pulverulina*, including two new species and we incorporated one species from *Porotheleum* into *Marasmiellomycena*. We also discovered three new species belonging to *Clitocybula*, *Gerronema* and *Leucoinocybe* in temperate and subtropical China. We accordingly present a morphological description of the new species and provide an identification key to the 22 species of Porotheleaceae currently known from China.

## ﻿Materials and methods

### ﻿Specimens and morphology

Macroscopic descriptions were based on the study of fresh specimens, whereas micromorphological descriptions relied on dried materials. In our descriptions, colour abbreviations follow the colour standards and colour nomenclature of Ridgway (1912). Microscopic observations were made on dried specimens mounted in 5% potassium hydroxide (KOH) and stained with Congo red when necessary. The prepared specimens were observed under a Lab A1 microscope (Carl Zeiss AG, Jena, Germany) and photographed and recorded using ZEN 2.3 software (Carl Zeiss AG). Melzer’s reagent was used to test whether spores and tissues were amyloid (Horak 2005). Twenty mature basidiospores from each basidiomata (two basidiomata per holotype) were measured in side view. Sizes of basidiospores were recorded, with the notation [*a/b/c*] used at the beginning of each entry in the description to indicate *a* basidiospores from *b* basidiomata of *c* specimens were measured. Measured sizes (including Q values) are given in the description as (d)e–f–g(h) × (i)j–k–l(m), where *d* is the smallest length, *e–g* represents the range of at least 90% of values, *f* is the average length and *h* is the largest value; width (*i–m*) is expressed in the same way. In addition, Q stands for the length-width ratio of a spore and Q ± av is the average Q of all basidiospores ± the sample standard deviation (Ge et al. 2021; Liu et al. 2021, 2022; Na et al. 2021, 2022a, 2022b). Hyphae of the pileipellis and stipitipellis and a total of 20 basidia, cheilocystidia and caulocystidia were measured from each collection. The examined collections have been deposited in the fungarium of the Fujian Academy of Agricultural Sciences (FFAAS), China. Author abbreviations follow Index Fungorum (http://www.indexfungorum.org).

### ﻿DNA extraction, polymerase chain reaction (PCR) amplification and sequencing

Genomic DNAs of the putative new species were extracted from dried materials using a NuClean PlantGen DNA kit (Kangwei Century Biotechnology Co., Beijing, China). Gene regions were amplified using the following primer pairs: ITS1/ITS4 (White et al. 1990) for 5.8S and internal transcribed spacer ITS1 and ITS2 regions (ITS), LR0R/LR7 (Hopple and Vilgalys 1999) for the large subunit of nuclear ribosomal DNA (nrLSU) and bRPB2-6f/bRPB2-7.1R (Matheny 2005) for the second largest subunit of RNA polymerase II (*rpb2*). Amplifications were performed in 25 µl reaction mixtures consisting of 9.5 μl ddH_2_O, 12.5 µl 2× UTaq PCR Master Mix (Zoman Biotechnology Co., Beijing, China), 1 µl of each primer (10 mM) and 1 μl DNA template. PCR amplification of the ITS region used the following protocol: initial denaturation at 95 °C for 4 min, followed by 34 cycles of 94 °C for 45 s, 52 °C for 45 s and 72 °C for 1 min and a final extension at 72 °C for 10 min. Cycling conditions used for amplification of the nrLSU were as follows: initial denaturation at 93 °C for 2 min, followed by 20 cycles of 93 °C for 1 min, 50 °C for 1 min and 72 °C for 1 min and a final extension at 72 °C for 10 min. The PCR protocol for *rpb2* amplification was as follows: initial denaturation at 93 °C for 2 min, 20 cycles of 93 °C for 1 min, 50 °C for 1 min and 72 °C for 1 min, 20 cycles of 93 °C for 1 min, 53 °C for 1 min and 72 °C for 1 min and a final extension at 72 °C for 10 min. The PCR products were subjected to Sanger dideoxy sequencing at the Beijing Genomics Institute (Beijing, China).

### ﻿Phylogenetic analysis

For phylogenetic analysis, we constructed a concatenated dataset of 168 ITS, 87 nrLSU and 14 *rpb2* sequences from 58 taxa of 14 genera of Porotheleaceae. In addition, six sequences (three ITS and three nrLSU) of *Mycenapurpureofusca* (Peck) Sacc. were included as outgroups according to the results of Na et al. (2022a). Sequences retrieved from GenBank and those obtained in this study are listed in Table 1. Alignments were performed in Mafft 7.376 (Katoh and Standley 2013). Sequence editing and necessary adjustments were carried out in BioEdit 7.0.4.1 and Clustal X 1.81 (Thompson et al. 1997; Hall 1999). Bayesian Inference (BI) and Maximum Likelihood (ML) bootstrap analyses were performed using the best-fit substitution models identified in ModelTest 3.7 (Posada and Crandall 1998). The BI analysis was carried out in MrBayes 3.2.6 (Ronquist and Huelsenbeck 2003). Runs of 1,000,000 generations, with trees sampled every 100^th^ generation, were initiated for eight heated and one cold Markov chain(s). Analyses were automatically terminated when the average standard deviation of split frequencies reached a value below 0.01 and the first 25% of trees were discarded as burn-in (Ronquist and Huelsenbeck 2003). The ML analysis was performed in RAxML GUI 2.0 using a rapid bootstrapping algorithm involving 1,000 replicates (Edler et al. 2021). The aligned datasets for Bayesian and ML analyses have been deposited in TreeBASE (submission ID 31062; study accession URL: http://purl.org/phylo/treebase/phylows/study/TB2:S31062). Phylogenetic trees were displayed using FigTree v.1.4.3.

**Table 1.** Specimens used in phylogenetic analysis, with geographic origin and GenBank accession numbers.

No.	Taxa	Voucher	Locality	ITS Sequence ID	LSU Sequence ID	rpb2 Sequence ID	Reference
1	*Chrysomycenaperplexa*	MCVE:30184 TYPE	Italy	NR172974	NG071251	–	Vizzini et al. (2019)
2	*Clitocybulaalbida*	CUH AM064	India	MG250188	–	–	Dutta et al. (2018)
3	*Clitocybulaalbida*	CUH AM065	India	MG250189	–	–	Dutta et al. (2018)
4	*Clitocybulaabundans*	STU:SMNS-B-FU-2017/00898	Germany	MF627833	–	–	Unpublished
5	*Clitocybulafamilia*	2319-QFB-25741	Canada	KM406970	–	–	Unpublished
6	*Clitocybulafamilia*	PRM 921866	Czech Republic	JF730327	JF730320	–	Antonín et al. (2011)
7	*Clitocybulafamilia*	BRNM 736053	Slovakia	JF730328	JF730323	–	Antonín et al. (2011)
8	*Clitocybulafamilia*	STU:SMNS-B-FU-2017/00926	Germany	MF627834	–	–	Unpublished
9	*Clitocybulafamilia*	NAMA 2017-349	USA	MH979253	–	–	Unpublished
10	***Clitocybulafuscostriata***	**FFAAS1029**	**China**	**OR238881**	**OR238893**	**OR258374**	**This study**
11	***Clitocybulafuscostriata***	**FFAAS1030 Holotype**	**China**	**OR238882**	**OR238894**	**OR258375**	**This study**
12	***Clitocybulafuscostriata***	**FFAAS1031**	**China**	**OR238883**	**OR238895**	**OR258376**	**This study**
13	*Clitocybulalacerata*	LE 6639	Russia	HM191746	–	–	Malysheva and Morozova (2011)
14	*Clitocybulalacerata*	LE 262744	Russia	HM191747	–	–	Malysheva and Morozova (2011)
15	*Clitocybulalacerata*	LE 262743	Russia	HM191748	–	–	Malysheva and Morozova (2011)
16	*Clitocybulalacerata*	PRM 915404	Czech Republic	LT854054	LT854030	–	Antonín et al. (2019)
17	*Clitocybulalacerata*	WU 19575	Austria	LT854053	LT854031	–	Antonín et al. (2019)
18	*Clitocybulaoculus*	3512	Canada	KM406971	–	–	Unpublished
19	*Clitocybulaoculus*	WU 20008	Canada	LT854017	LT854017	–	Antonín et al. (2019)
20	*Clitocybulaoculus*	S.D. Russell iNaturalist # 8606755	India	MN906165	–	–	Unpublished
21	*Clitocybulaoculus*	S.D. Russell iNaturalist # 8591258	India	MN906164	–	–	Unpublished
22	*Clitocybulaoculus*	BIOUG24046-B03	Canada	KT695321	–	–	Telfer et al. (2015)
23	*Clitocybulaoculus*	AFTOL-ID 1554	USA	DQ192178	DQ192178	–	Matheny et al. (2006)
24	*Delicatulaintegrella*	KA12-1305	Korea	KR673538	–	–	Kim et al. (2015)
25	*Delicatulaintegrella*	S.D. Russell MycoMap # 6067	USA	MN906231	–	–	Unpublished
26	*Delicatulaintegrella*	G0060	USA	–	MK277924	–	Varga et al. (2019)
27	*Gerronemabaishanzuense*	FFAAS0359 Holotype	China	OL985962	OL985984	–	Na et al. (2022a)
28	*Gerronemabaishanzuense*	FFAAS0360	China	OL985963	–	–	Na et al. (2022a)
29	*Gerronemabaishanzuense*	FFAAS0361	China	OL985964	OL985985	–	Na et al. (2022a)
30	*Gerronemabaishanzuense*	FFAAS0362	China	OL985965	OL985986	–	Na et al. (2022a)
31	*Gerronemabaishanzuense*	FFAAS0363	China	OL985966	OL985987	–	Na et al. (2022a)
32	*Gerronemabaishanzuense*	FFAAS0366	China	OL985967	OL985988	–	Na et al. (2022a)
33	***Gerronemabrunneosquamulosum***	**FFAAS1032 Holotype**	**China**	**OR238884**	**OR238896**	**OR258377**	**This study**
34	***Gerronemabrunneosquamulosum***	**FFAAS1033**	**China**	**OR238885**	**OR238897**	**OR258378**	**This study**
35	*Gerronemaindigoticum*	HMJAU 47636	China	MK693727	MK693732	–	Liu et al. (2019)
36	*Gerronemaindigoticum*	HMJAU 47942	China	MK693728	MK693733	–	Liu et al. (2019)
37	*Gerronemaindigoticum*	HMJAU 47943	China	MK693729	MK693734	–	Liu et al. (2019)
38	*Gerronemakeralense*	2	India	MH156555	NG_064531	–	Latha et al. (2018)
39	*Gerronemakeralense*	BKF10263	Thailand	MZ452107	MZ452144		Direct Submission
40	*Gerronemakuruvense*	CAL 1665	India	NG_159831	NG_064530	–	Latha et al. (2018)
41	*Gerronemakuruvense*	BKF10266	Thailand	MZ452090	MZ452669	–	Direct Submission
42	*Gerronemakuruvense*	DCY3362(HGASMF01-15010)	Chian	MZ951144	–	–	Direct Submission
43	*Gerronemamicrocarpum*	FFAAS0365	China	–	OL985989	–	Na et al. (2022a)
44	*Gerronemamicrocarpum*	FFAAS0371	China	OL985968	OL985990	–	Na et al. (2022a)
45	*Gerronemamicrocarpum*	FFAAS0372	China	OL985969	OL985991	–	Na et al. (2022a)
46	*Gerronemamicrocarpum*	FFAAS0373 Holotype	China	OL985970	OL985992	–	Na et al. (2022a)
47	*Gerronemamicrocarpum*	FFAAS0374	China	OL985971	–	–	Na et al. (2022a)
48	*Gerronemamicrocarpum*	FFAAS0375	China	OL985972	OL985993	–	Na et al. (2022a)
49	*Gerronemanemorale*	KACC 43599	Korea	EU883592	–	–	Unpublished
50	*Gerronemanemorale*	KACC 43600	Korea	EU883593	–	–	Unpublished
51	*Gerronemanemorale*	not indicated	Korea	EU883594	–	–	Unpublished
52	*Gerronemanemorale*	FA249	Pakistan	MN744686	–	–	Aqdus and Khalid (2021)
53	*Gerronemanemorale*	FA236	Pakistan	MN744687	–	–	Aqdus and Khalid (2021)
54	*Gerronemanemorale*	FA239	Pakistan	MN744688	–	–	Aqdus and Khalid (2021)
55	*Gerronemastrombodes*	DJL05NC72	USA	EU623639	–	–	Hughes et al. (2007)
56	*Gerronemastrombodes*	TFB12519/TENN60718	USA	EU623640	–	–	Hughes et al. (2007)
57	*Gerronemastrombodes*	TFB12783/TENN61350	USA	EU623641	–	–	Hughes et al. (2007)
58	*Gerronemastrombodes*	TFB11947 clone C2	USA	KY242503	–	–	Hughes et al. (2007)
59	*Gerronemastrombodes*	TFB11947 clone C3	USA	KY242504	–	–	Hughes et al. (2007)
60	*Gerronemastrombodes*	TFB11947 clone C5	USA	KY242506	–	–	Hughes et al. (2007)
61	*Gerronemastrombodes*	TFB14234	USA	KY242507	–	–	Hughes et al. (2007)
62	*Gerronemastrombodes*	TFB14514	USA	KY242509	–	–	Hughes et al. (2007)
63	*Gerronemastrombodes*	TFB11947	USA	KY271083	–	–	from GenBank
64	*Gerronemasubclavatum*	Redhead 5175, DAOM	not indicated	U66434	–	–	Lutzoni (1997)
65	*Gerronemasubclavatum*	FLAS-F-60986	USA	MH016932	–	–	from GenBank
66	*Gerronemasubclavatum*	FLAS-F-61518	USA	MH211945	–	–	from GenBank
67	*Gerronemasubclavatum*	Smith-2018	USA	MK573888	–	–	Direct Submission
68	*Gerronemasubclavatum*	Mushroom Observer # 243440	USA	MK607510	–	–	Direct Submission
69	*Gerronemasubclavatum*	iNaturalist # 8545787	India	MN906021	–	–	from GenBank
70	*Gerronemasubclavatum*	S.D. Russell MycoMap # 6854	India	MN906138	–	–	from GenBank
71	*Gerronemawaikanaense*	PDD:87667	New Zealand	JQ694117	–	–	from GenBank
72	*Gerronemawildpretii*	BRNM 788347	Madeira	LT854045	LT854043	–	Antonin et al. (2019)
73	*Gerronemaxanthophyllum*	PRM 924657	Czech Republic	LT854023	LT854023	–	Antonin et al. (2019)
74	*Gerronemazhujian*	FFAAS0364	China	OL985973	OL985994	–	Na et al. (2022a)
75	*Gerronemazhujian*	FFAAS0370	China	OL985974	OL985995	–	Na et al. (2022a)
76	*Gerronemazhujian*	FFAAS0376 Holotype	China	OL985975	OL985996	–	Na et al. (2022a)
77	*Hydropodiasubalpina* (≡*Hydropussubalpinus*)	STU:SMNS-STU-F-0900123	Germany	MF039248	–	–	Eberhardt et al. (2018)
78	*Hydropodiasubalpina* (≡*Hydropussubalpinus*)	Montri-291	not indicated	MK028414	–	–	Unpublished
79	*Hydropodiasubalpina* (≡*Hydropussubalpinus*)	Montri-312	not indicated	MK028415	–	–	Unpublished
80	*Hydropodiasubalpina* (≡*Hydropussubalpinus*)	Montri-323	not indicated	MK028416	–	–	Unpublished
81	*Hydropodiasubalpina* (≡*Hydropussubalpinus*)	OKA-TR-K364	Turkey	MN701620	MN700170	–	Unpublished
82	*Hydropodiasubalpina* (≡*Hydropussubalpinus*)	OKA-TR-K380	Turkey	MN701621	MN700171	–	Unpublished
83	*Hydropodiasubalpina* (≡*Hydropussubalpinus*)	OKA-TR-B400	Turkey	MN701622	MN700172	–	Unpublished
84	*Hydropusatramentosus*	918	Italy	JF908050	–	–	Osmundson et al. (2013)
85	*Hydropusfuliginarius*	S.D. Russell ONT iNaturalist # 130794969	USA	OP643427	–	–	Unpublished
86	*Hydropusfuliginarius*	DAOM196062	USA	–	AF261368	–	Moncalvo et al. (2002)
87	*Hydropusmarginellus*	AFTOL-ID 1720	not indicated	DQ490627	DQ457674	DQ472722	Matheny et al. (2006)
88	*Hydropusmarginellus*	OSC 112834	USA	EU669314	EU852808	–	Unpublished
89	*Hydropusrugosodiscus*	MGW1257	USA	KY777386	–	–	Unpublished
90	*Hydropusrugosodiscus*	PBM4022	USA	KY777390	–	–	Unpublished
91	*Hydropusrugosodiscus*	Taxon 10	not indicated	MW399385	–	–	Unpublished
92	*Leucoinocybeauricoma* (≡*Mycenaauricoma*)	HKAS126433	China	OQ025169	–	–	Direct Submission
93	*Leucoinocybeauricoma* (≡*Mycenaauricoma*)	AFTOL-ID 1341 (specimen_voucher HKAS41510)	China	DQ490647	–	–	Matheny et al. (2006)
94	*Leucoinocybedanxiashanensis*	GDGM79543	China	MZ667475	MZ667479	–	Unpublished
95	*Leucoinocybedanxiashanensis*	GDGM80113	China	MZ667476	MZ667480	–	Unpublished
96	*Leucoinocybedanxiashanensis*	GDGM80114	China	MZ667477	MZ667481	–	Unpublished
97	*Leucoinocybedanxiashanensis*	GDGM80184	China	MZ667478	MZ667482	–	Unpublished
98	*Leucoinocybeflavoaurantia*	D	Italy	HM191743	–	–	Malysheva and Morozova (2011)
99	*Leucoinocybeflavoaurantia*	GDOR	Italy	HM191744	–	–	Malysheva and Morozova (2011)
100	*Leucoinocybeflavoaurantia*	LE 262757	Russia	HM191745	–	–	Malysheva and Morozova (2011)
101	*Leucoinocybelenta*	BOZ (EPITYPE)	Italy	–	LT854032	–	Antonín et al. (2019)
102	*Leucoinocybelishuiensis*	FFAAS 0111 (HOLOTYPE)	China	MW424488	MW424492	–	Na et al. (2021)
103	*Leucoinocybelishuiensis*	FFAAS 0112	China	MW424489	MW424493	–	Na et al. (2021)
104	*Leucoinocybelishuiensis*	FFAAS 0113	China	MW424490	MW424494	–	Na et al. (2021)
105	*Leucoinocybelishuiensis*	FFAAS 0115	China	MW424491	MW424495	–	Na et al. (2021)
106	*Leucoinocybe* sp.	KA12-0435	South Korea	KR673482	–	–	Kim et al. (2015)
107	***Leucoinocybesubglobispora***	**FFAAS1034 Holotype**	**China**	**OR238886**	**OR238898**	**OR258379**	**This study**
108	***Leucoinocybesubglobispora***	**FFAAS1035**	**China**	**OR238887**	**OR238899**	**OR258380**	**This study**
109	*Leucoinocybesulcata*	CAL 1246 (HOLOTYPE)	India	KR029720	KR029721	–	Latha et al. (2015)
110	*Leucoinocybetaniae*	BCN-SCM B-4064	Italy	LT854057	LT854028	–	Antonín et al. (2019)
111	*Marasmiellomycenaalbodescendens*	PDD 96142	New Zealand	OL998341	OL998380	–	Consiglio et al. (2022)
112	*Marasmiellomycenaalbodescendens*	PDD 96321	New Zealand	OL998343	OL998382	–	Consiglio et al. (2022)
113	*Marasmiellomycenaomphaliiforme* (≡*Porotheleumomphaliiforme*)	WU 16775	Italy	OM422777	OM423654	–	Direct Submission
114	*Marasmiellomycenaomphaliiforme* (≡*Porotheleumomphaliiforme*)	LIP 0401689	France	OM422780	OM423655	–	Direct Submission
115	*Marasmiellomycenaomphaliiforme* (≡*Porotheleumomphaliiforme*)	AMB 18850	France	OM422781	OM423656	–	Direct Submission
116	*Marasmiellomycenaomphaliiforme* (≡*Porotheleumomphaliiforme*)	AMB 18845	France	OM422782	–	–	Direct Submission
117	*Marasmiellomycenapseudoomphaliiformis*	BRNM:552721	USA	OR913562	OR913566	–	Senanayake et al. (2023)
118	*Marasmiellomycenapseudoomphaliiformis*	BRNM:552654	USA	OR913560	OR913564	–	Senanayake et al. (2023)
119	*Marasmiellomycenapseudoomphaliiformis*	BRNM:552658	USA	OR913561	OR913565	–	Senanayake et al. (2023)
120	***Marasmiellomycenatomentosa***	**FFAAS1036 Holotype**	**China**	**OR238888**	**OR238900**	**OR258381**	**This study**
121	***Marasmiellomycenatomentosa***	**FFAAS1037**	**China**	**OR238889**	**OR238901**	**OR258382**	**This study**
122	***Marasmiellomycenatomentosa***	**FFAAS1038**	**China**	**OR238890**	**OR238902**	**OR258383**	**This study**
123	*Megacollybiaclitocyboidea*	TFB11884/TENN60766	USA	EU623658	–	–	Hughes et al. (2007)
124	*Megacollybiaclitocyboidea*	TENN62231	USA	EU623664	–	–	Hughes et al. (2007)
125	*Megacollybiaclitocyboidea*	TENN62230 clone c4	USA	EU623673	–	–	Hughes et al. (2007)
126	*Megacollybiaclitocyboidea*	TENN62230 clone c5	USA	EU623674	–	–	Hughes et al. (2007)
127	*Megacollybiafallax*	MICH 45002	USA	EU623714	–	–	Hughes et al. (2007)
128	*Megacollybiafallax*	TFB11561/TENN59447	USA	EU623723	–	–	Hughes et al. (2007)
129	*Megacollybiafallax*	DAOM208710	USA	EU623724	–	–	Hughes et al. (2007)
130	*Megacollybiafallax*	Mushroom Observer 291302	USA	MN176984	–	–	Direct Submission
131	*Megacollybiafallax*	Mushroom Observer 286893	USA	MT437075	–	–	Direct Submission
132	*Megacollybiamarginata*	PRM 860926	Czech Republic	LT854022	–	–	Antonín et al. (2019)
133	*Megacollybiamarginata*	PRM 859785	Czech Republic	LT854046	LT854042	–	Antonín et al. (2019)
134	*Megacollybiamarginata*	HR 91642	Czech Republic	LT854050	–	–	Antonín et al. (2019)
135	*Megacollybiamarginata*	HR 91607	Czech Republic	LT854051	–	–	Antonín et al. (2019)
136	*Megacollybiaplatyphylla*	AFTOL-ID 560	USA	DQ249275	AY635778	DQ385887	Unpublished
137	*Megacollybiaplatyphylla*	BRNM 737654	Czech Republic	LT854048	LT854036	–	Antonín et al. (2019)
138	*Megacollybiaplatyphylla*	BRNM 766972	Czech Republic	LT854049	LT854037	–	Antonín et al. (2019)
141	*Megacollybiarodmani*	BHS2009-06	USA	GQ397989	–	–	from GenBank
149	*Megacollybiarodmani*	PUL F27039	USA	MW448576	–	–	from GenBank
150	*Megacollybiasubfurfuracea*	TFB11075/TENN59558 clone c3	USA	EU623744	–	–	Hughes et al. (2007)
151	*Megacollybiasubfurfuracea*	TFB11075/TENN59558 clone c8	USA	EU623745	–	–	Hughes et al. (2007)
152	*Megacollybiatexensis*	DPL7405/TENN62058 clone c1	USA	EU623725	–	–	Hughes et al. (2007)
153	*Megacollybiatexensis*	DPL7405/TENN62058 clone c2	USA	EU623726	–	–	Hughes et al. (2007)
154	*Megacollybiatexensis*	FLAS-F-61511	USA	MH211940	–	–	from GenBank
155	*Mycenapurpureofusca*	HMJAU 43554	China	MG654740	MK629356	–	Na and Bau (2018)
156	*Mycenapurpureofusca*	HMJAU 43624	China	MG654741	MK629357	–	Na and Bau (2018)
157	*Mycenapurpureofusca*	HMJAU 43640	China	MG654742	MK629358	–	Na and Bau (2018)
158	*Porotheleumfimbriatum*	Dai 12276	China	KX081137	KX161656	–	from GenBank
159	*Porotheleumfimbriatum*	Dai 12289	China	KX081138	KX161654	–	from GenBank
160	*Porotheleumfimbriatum*	CLZhao 1120	China	MH114870	–	–	from GenBank
161	*Porotheleumfimbriatum*	CLZhao 2368	China	MH114871	–	–	from GenBank
162	*Porotheleumfimbriatum*	SWFC 006350	China	MK894078	–	–	from GenBank
163	*Porotheleumfimbriatum*	SWFC 006399	China	MK894079	–	–	from GenBank
164	*Porotheleumparvulum*	JBSD131802 Type	Dominican Republic	NR_182714	OM423657	–	Consiglio et al. (2022)
165	*Pseudohydropusfloccipes*	AMB 18768	Spain	–	OM423637	–	Consiglio et al. (2022)
166	*Pseudohydropusfloccipes*	BRNM 825631	Spain	OM422760	OM423636	–	Consiglio et al. (2022)
167	*Pseudohydropusfloccipes*	BRNM 751633	Spain	OM422759	OM423635	–	Consiglio et al. (2022)
168	*Pseudohydropusglobosporus*	BAP 661 (Holotype, SFSU)	USA	OM422758	OM423634	–	Cooper et al. (2019)
169	*Pseudohydropus* sp	MushroomObserver490861	Jamaica	OR879917	–	–	Direct Submission
170	***Pulverulinaflavoalba***	**FFAAS1039 Holotype**	**China**	**OR238891**	**OR238903**	**OR258384**	**This study**
171	***Pulverulinaflavoalba***	**FFAAS1040**	**China**	**OR238892**	**OR238904**	**OR258385**	**This study**
172	*Pulverulinaulmicola*	TENN 029208 Holotype	USA	NR_119887	HQ179668	–	Matheny et al. (2020)
173	*Pulverulinaulmicola*	TFB13871	USA	MT237476	MT237446	–	Matheny et al. (2020)
174	*Pulverulinaulmicola*	KUBOT-KRMK-2020-13	India	MW425325	MW425344	–	Unpublished
175	*Trogiabenghalensis*	CUH AM031	India	KU647630	–	–	Dutta et al. (2017)
176	*Trogiabenghalensis*	CUH AM122	India	MF967246	–	–	Dutta et al. (2017)
177	*Trogiainfundibuliformis*	KUN_HKAS63661	China	JQ031775	JQ031780	–	Yang et al. (2012)
178	*Trogiainfundibuliformis*	KUN_HKAS56709	China	JQ031776	JQ031781	–	Yang et al. (2012)
179	*Trogiavenenata*	KUN_HKAS54710	China	JQ031772	JQ031778	–	Yang et al. (2012)
180	*Trogiavenenata*	KUN_HKAS56679	China	JQ031773	JQ031779	–	Yang et al. (2012)
181	*Trogiavenenata*	TC2-28	China	KT968080	–	–	Mi et al. (2016)
182	*Trogiavenenata*	MHHNU 8750	China	KX268227	–	–	Unpublished
183	*Vizziniadomingensis* (≡*Porotheleumdomingense*)	JBSD131801a	Dominican Republic	OM422768	OM423646	–	Consiglio et al. (2022)
184	*Vizzinianigripes* (≡*Porotheleumnigripes*)	JBSD131803	Dominican Republic	OM422771	OM423648	–	Consiglio et al. (2022)

Note: Newly-generated sequences are in bold.

## ﻿Results

### ﻿Phylogenetic analysis

**Figure 1.**
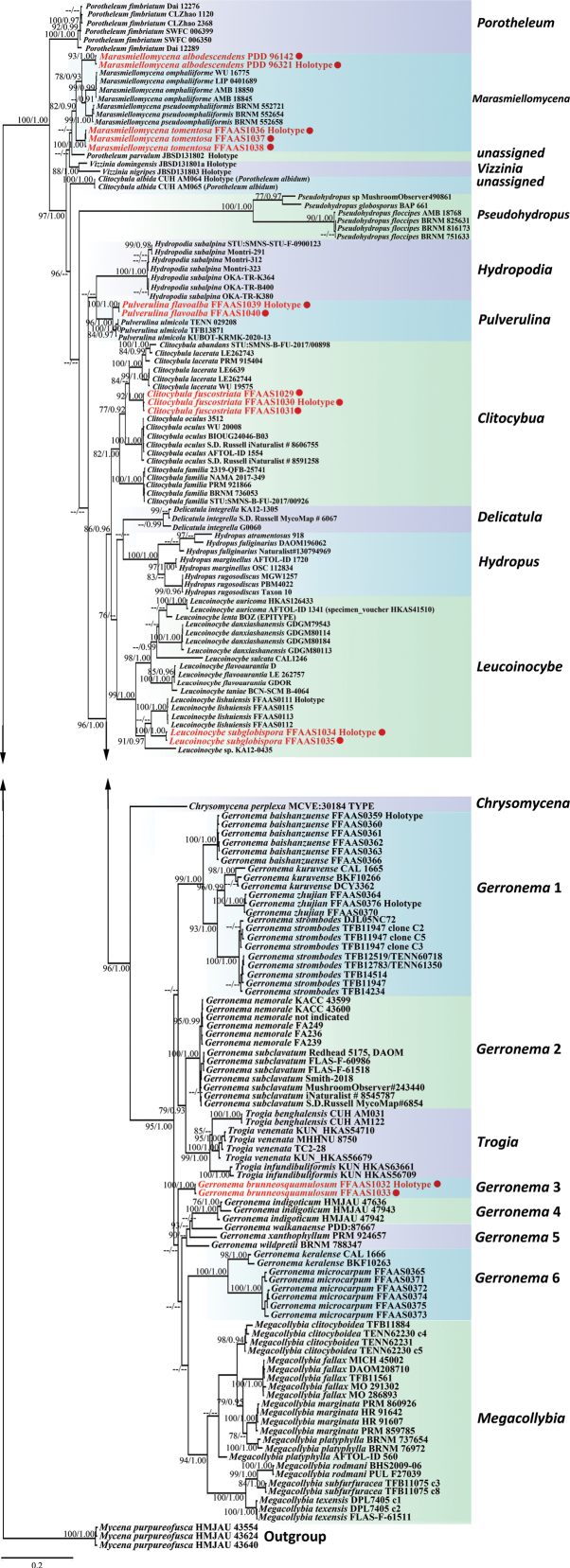
Phylogenetic consensus tree inferred from the Maximum Likelihood (ML) analysis based on a concatenated ITS, nrLSU and *rpb2* dataset (MLB ≥ 75%, BPP ≥ 0.90 are indicated). The tree is rooted with *Mycenapurpureofusca*. The new species and combination are marked by red.

A data matrix was created for 59 taxa, including 58 taxa of Porotheleaceae and, as an outgroup, one taxon of *Mycena*. Including gaps, the aligned dataset comprised 2,274 nucleotide sites: 974 for ITS, 610 for nrLSU and 690 for *rpb2* exons (all sites without introns). For the ML analysis, the best-fit substitution models selected for ITS, nrLSU and *rpb2*-exon partitions in the concatenated dataset were TPM2uf+I+G4, GTR +I+G4 and TIM2+I+G4, respectively. For the BI analysis, the best-fit substitution model selected for each of the three DNA regions (ITS, nrLSU and *rbp2* exons) was GTR+I+G. Phylogenetic reconstructions, based on BI and ML methods, yielded similar topologies. The BI topology was, therefore, selected as a representative phylogeny (Fig. 1).

In the tree shown in Fig. 1, 21 major well-supported clades are evident: *Chrysomycena*, *Clitocybula*, *Delicatula*, *Hydropodia*, *Hydropus*, *Leucoinocybe*, *Marasmiellomycena*, *Megacollybia*, *Pulverulina*, *Pseudohydropus*, *Trogia* and *Vizzinia*, all of which form monophyletic groups at the generic level. However, within *Porotheleum*, two species, totalling three specimens, form two unassigned clades. In addition, *Marasmiellomycena*, forms a well-supported (MLB = 81%; BPP = 0.90) independent clade comprising four species distinct from *Vizzinia* and the unassigned *Porotheleum*. In the phylogenetic tree, *Chrysomycena*, *Gerronema*, *Hydropus*, *Leucoinocybe*, *Megacollybia*, *Trogia* and five taxa of *Clitocybula* cluster together with high statistical support (MLB = 96%; BPP = 1.00), but one sequence of *Delicatula* appears outside this large clade in the Maximum Likelihood analysis. The variation in the phylogenetic analysis outcomes for *Delicatula* specimens can be ascribed to inconsistent sequence coverage. Of the three *Delicatula* specimens evaluated, two only contained ITS sequences clustered together into a clade (MLB = 99%; BPP = 0.68), suggesting some degree of relatedness. In contrast, the remaining specimen, which only included an LSU sequence, was placed differently across the analyses. Such disparities in sequence coverage are likely to be responsible for the observed discrepancies between different computational algorithms used in the phylogenetic reconstructions. *Hydropodia* and *Pulverulina*form a large, poorly supported clade. Moreover, *Hydropus* (MLB = 100%; BPP = 1.00), *Leucoinocybe* (MLB = 99%; BPP = 1.00) and *Clitocybula* (MLB = 82%; BPP = 1.00) are strongly supported as distinct genera and collectively constitute a distinct clade separate from all other clades. However, *Gerronema* is polyphyletic (*Gerronema* 1 to *Gerronema* 6), with each individual *Gerronema* clade sister to *Megacollybia* or *Trogia*. Finally, *Chrysomycena* and *Hydropodia* comprise a single species each.

In the phylogenetic tree, samples of the new species and new combination are placed in *Marasmiellomycena*, where they constitute monophyletic lineages, each with high statistical support (*M.albodescendens*: MLB = 93%, BPP = 1.00; *M.tomentosa*: MLB = 100%, BPP = 1.00). The four other new species are strongly supported as members of *Gerronema* 3, *Pulverulina*, *Leucoinocybe* and *Clitocybula* clades (*C.fuscostriata*: MLB = 92%, BPP = 1.00; *G.brunneosquamulosum*: MLB = 100%, BPP = 1.00; *L.subglobispora*: MLB = 100%, BPP = 1.00; and *Pulverulinaflavoalba*: MLB = 100%, BPP = 1.00). *Marasmiellomycenatomentosa* is closely related to a clade containing two species and a new combination, *M.albodescendens*, *M.omphaliiforme* and *M.pseudoomphaliiformis*. *Pulverulinaflavoalba* sp. nov. is grouped with high statistical support (MLB = 100%; BPP = 1.00) with three sequences of *Pulverulinaulmicola* (H.E. Bigelow) Matheny & K.W. Hughes from India and the USA (including holotype voucher no. TENN 029208). Within the *Leucoinocybe* clade, *L.subglobispora* constitutes a monophyletic lineage that is most closely related to *Leucoinocybelishuiensis*, a new species recently described from China (Na et al. 2021). *Clitocybulafuscostriata* is placed along with *C.lacerata* (Scop.) Métrod in an unresolved lineage that is treated as *C.lacerata* agg. by Antonín et al. (2019) and in our studies.

*Clitocybulaalbida* A.K. Dutta, K. Acharya & Antonín, reported from India as a new species, was transferred to *Porotheleum* [as *Porotheleumalbidum* (A.K. Dutta, K. Acharya & Antonín) Vizzini & Consiglio] and *Porotheleumparvulum* Angelini, Vizzini, Consiglio & M. Marchetti as a new species from the Dominican Republic (Dutta et al. 2018; Consiglio et al. 2022). The phylogenetic status of *Clitocybulaalbida* is currently unclear and treated as unassigned clades in the study of Senanayake et al. (2023). On the other hand, *Porotheleumparvulum* is known to cluster with *Marasmiellomycena* and *Vizzinia*, forming a clade. Within this clade, *Porotheleumparvulum* is specifically determined to be a sister group to *Marasmiellomycena*. In the research conducted by Senanayake et al. (2023), *Pseudohydropus* and *Pulverulina* were identified as forming a monophyletic group. Contrastingly, in our phylogenetic tree, *Pseudohydropus* emerges as an independent lineage, receiving robust support (MLB =100%; BPP = 1.00) and not aligning as a sister group with any other genera. The observed differences might stem from variances in sequence coverage and the evolutionary rates of the genes. While Senanayake et al. (2023) utilised ITS and LSU sequences for their phylogenetic construction, our study encompassed ITS, LSU and RPB2 in the combined phylogenetic analysis. (Fig. 1).

### ﻿Taxonomy

#### ﻿ Clitocybula fuscostriata

Taxon classificationFungiAgaricalesPorotheleaceae

Q.Na & Y.P.Ge sp. nov.

00A8A2B2-A52A-510D-AF72-1D4FA3C5CA26

849407

Figs 2 , 3 , 4

##### Diagnosis.

Pileus with dark-brown striae. Differs from *C.striata* in having broader basidiospores and lacking hymenial cystidia.

**Figure 2.**
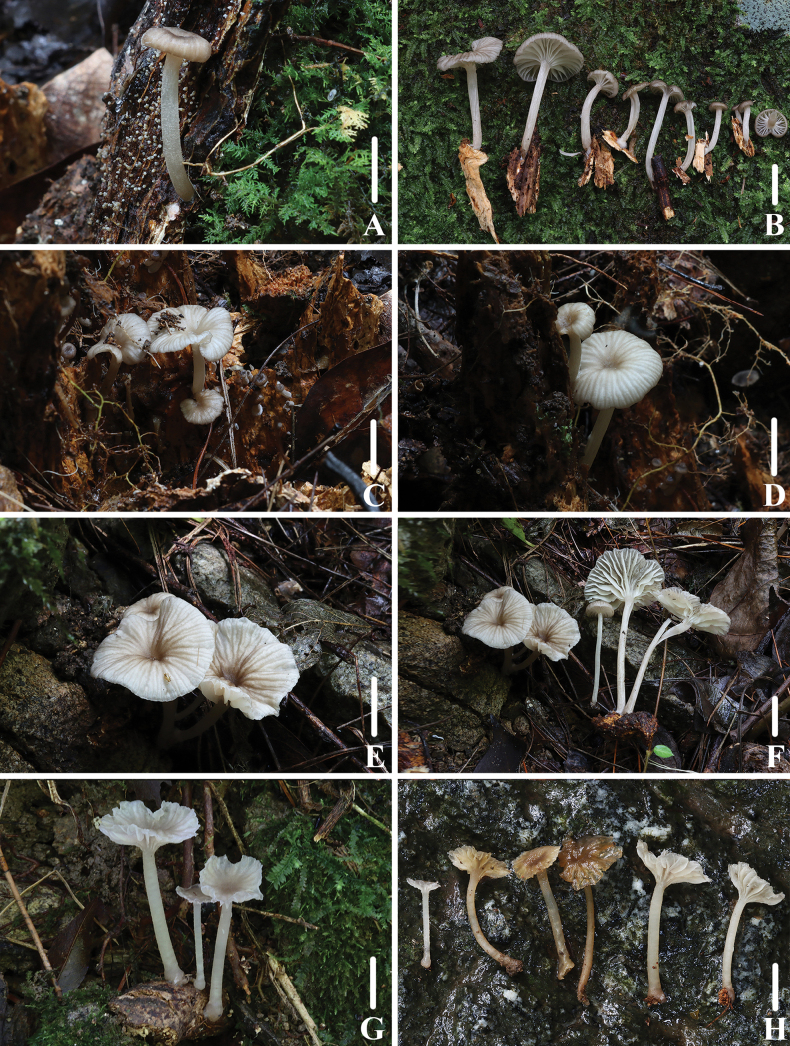
Basidiomata of *Clitocybulafuscostriata***A–D** collection *FFAAS1029***E–F** collection *FFAAS1030*, holotype **G–H** collection *FFAAS1031*. Scale bars: 10 mm (**A–H**).

##### Holotype.

China. Zhejiang Province: Baiyun National Forest Park, Liandu District, Lishui City, 2 Aug 2021, Qin Na, Yupeng Ge, Zewei Liu, Yaping Hu, Changjing Liu and Hui Ding, *FFAAS1030* (collection number MY0460).

**Figure 3.**
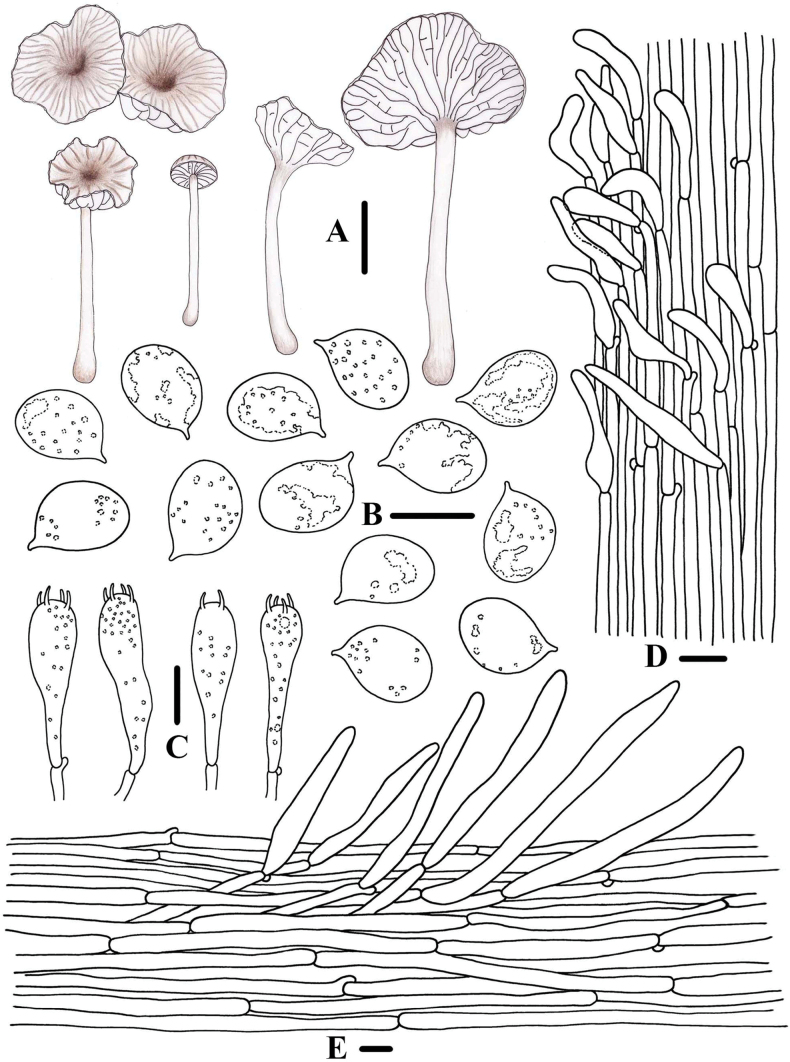
Morphological features of *Clitocybulafuscostriata* (*FFAAS1030*, holotype) **A** basidiomata **B** basidiospores **C** basidia **D** caulocystidia **E** pileipellis and pileocystidia. Scale bars: 10 mm (**A**); 5 μm (**B**); 10 μm (**C–E**).

##### Etymology.

Name refers to the pileus with radially fuscous striae.

**Figure 4.**
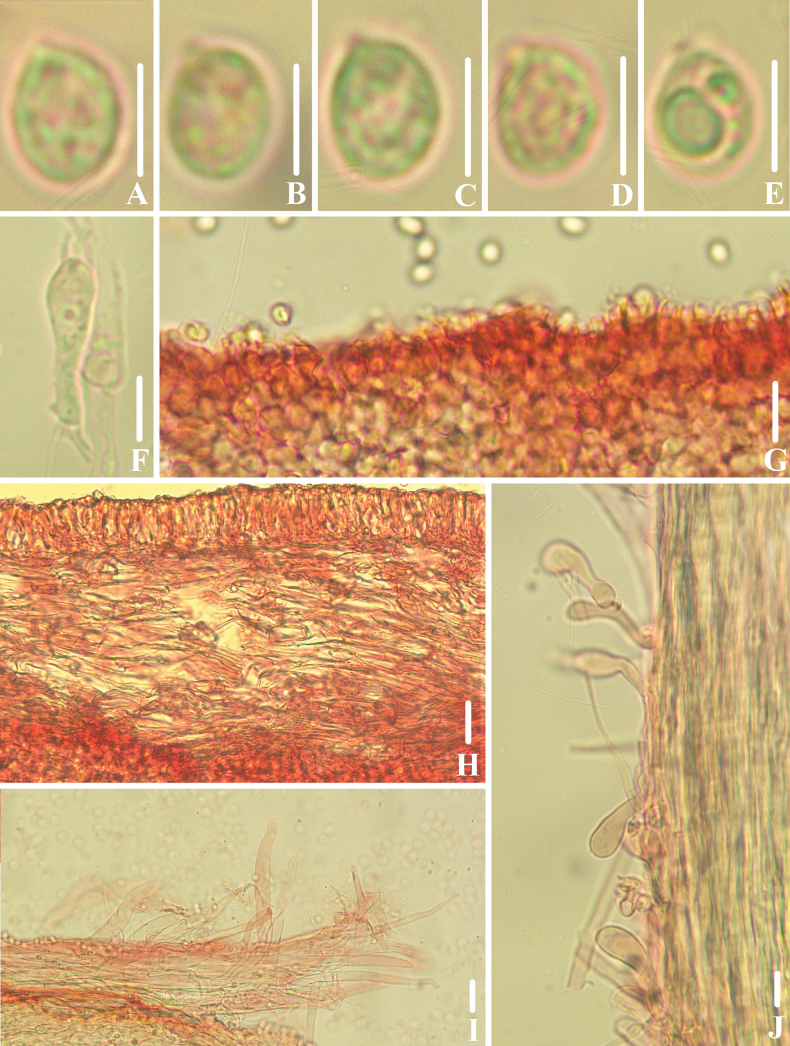
Microscopic features of *Clitocybulafuscostriata* (*FFAAS1030*, holotype) **A–E** basidiospores **F** basidia **G** margin of lamellae **H** lamellar trama **I** pileipellis and pileocystidia **J** caulocystidia. Scale bars: 5 μm (**A–E**); 10 μm (**F–J**). Structures were stained with 1% Congo Red aqueous solution before photographing.

##### Description.

Pileus 3.0–28.5 mm in diameter, hemispherical at first, then convex with depressed centre, expanded with age, infundibuliform with deeply umbilicate at the centre when old, thin-fleshed, dry, surface innately radially Fuscous (XLVI13′′′′k) to Fuscous-Black (XLVI13′′′′m) striate, surface somewhat fibrillose, becoming glabrous, radially cracked at margin when old, Benzo Brown (XLVI13′′′′i), Hair Brown (XLVI17′′′′i), Fuscous (XLVI13′′′′k) to Fuscous-Black (XLVI13′′′′m) at the centre, Pale Smoke Grey (XLVI21′′′′f) in the margin when young, Pale Smoke Grey (XLVI21′′′′f) to Smoke Grey (XLVI21′′′′d) with Bone Brown (XL13′′′m) at the centre when old. Context thin, white, fragile. Lamellae subdecurrent, white, with 1–3 tiers of lamellulae, irregularly intervenose, edges concolorous with the face. Stipe 17.0–52.0 × 1.0–2.5 mm, hollow, cylindrical, strongly and coarsely grooved, slightly bulbous at the base, fragile, finely whitish fibrillose, white in the upper part, Citrine Drab (XL21′′′i) in the base, base covered with a few white fibrils. Odour and taste inconspicuous.

Basidiospores (80/4/3) (5.2) 5.4–5.8–6.2 (6.5) × (4.2) 4.3–4.7–5.0 (5.1) μm [Q = 1.13–1.34, Q = ***1.25*** ± 0.050] [holotype (40/2/1) (5.3) 5.5–5.8–6.2 (6.5) × (4.2) 4.4–4.6–5.0 (5.1) μm, Q = 1.17–1.32, Q = *1.26* ± 0.040], broadly ellipsoid, hyaline in 5% KOH, smooth, thin-walled, guttulate, amyloid. Basidia 22–32 × 5–9 μm, 2- or 4-spored, clavate, sterigmata 2.5–4.7 × 0.6–1.6 μm. Hymenial cystidia absent. Lamellae edge cells scattered, cylindrical, narrowly clavate, thin-walled. Lamellar trama subregular; hyphae 3–7 μm wide, thin-walled, hyaline, non-dextrinoid. Pileipellis hyphae 4–9 μm wide, smooth; pileocystidia 70–162 × 7–19 μm, cylindrical or narrowly clavate, apically obtuse, thin-walled, hyaline, smooth. Stipitipellis a cutis made up of 3–8 μm wide hyphae, smooth, thin-walled; caulocystidia 27–63 × 5–8 μm, cylindrical, clavate, fusoid, apically obtuse, thin-walled base, smooth, transparent. Clamps present in all tissues.

##### Habit and habitat.

Scattered on rotten branches or twigs in *Acer*, *Armeniaca*, *Cercidiphyllum*, *Emmenopterys* and *Picea* mixed forests.

##### Known distribution.

Zhejiang Province, China.

##### Additional material examined.

China. Zhejiang Province: Baiyun National Forest Park, Liandu District, Lishui City, 2 Aug 2021, Qin Na, Yupeng Ge, Hui Zeng and Yulan Sun, *FFAAS1029* (collection number MY0459); Zhejiang Province: Baiyun National Forest Park, Liandu District, Lishui City, 2 Aug 2021, Qin Na, Yupeng Ge, Zewei Liu, Yaping Hu, Changjing Liu and Hui Ding, *FFAAS1031* (collection number MY0466).

##### Notes.

*Clitocybulafuscostriata* is considered to be a distinct species in the genus on account of its pileus with dark-brown striae, broadly ellipsoid basidiospores, absence of cheilocystidia and pleurocystidia and thin-walled pileipellis and stipitipellis hyphae. Five recorded species morphologically resemble this new species: *C.familia* (Peck) Singer, *C.lacerata* (Scop.) Métrod, *C.oculata* (Murrill) H.E. Bigelow, *C.striata* Dähncke, Contu & Vizzini and *C.tilieti* (Singer) Singer (Singer 1943; Romagnesi 1968; Bigelow 1973; Lennox 1979; Ludwig 2000, 2001; Dähncke et al. 2010; Antonín et al. 2011). *Clitocybulastriata*, a new taxon reported from Spain, has certain morphological similarities to *C.fuscostriata*, namely, a grey-brown to brown pileus with dark-brown striae, but differs from *C.striata* in having ellipsoid basidiospores (5–7 × 3.5–4.8 μm; Q = 1.5) and presence of utriform or lageniform cheilocystidia (Dähncke et al. 2010). In contrast to *C.fuscostriata*, *C.tilieti* can be easily mistaken for *C.striata*, but the pileus of *C.tilieti* is distinctly viscid and its stipitipellis and caulocystidia are thick-walled (Singer 1943; Antonín et al. 2011). *Clitocybulalacerata* (Scop.) Métrod, the type species of *Clitocybula*, is characterised by its caespitose stipes, beige-grey to pale-grey brown pileus, presence of clavate cheilocystidia and a pileipellis with pale encrusting pigmentation, differentiating it from *C.fuscostriata* (Peck 1878; Breitenbach and Kranzlin 1991; Ludwig 2000, 2001; Antonín et al. 2019). *Clitocybulaoculata* (Murrill) H.E. Bigelow and *C.familia* resemble *C.fuscostriata* in colour and size of the pileus and stipe, but can be distinguished from the new species by the size and shape of the basidiospores [*C.oculata* basidiospores (8.5–)10–12(–13) × 6–9 μm, broadly ellipsoid or ovate; *C.familia* basidiospores 3.5–5.3(–5.5) × 3.5–5.0 μm, globose, subglobose to broadly ellipsoid] (Romagnesi 1968; Bigelow 1973; Lennox 1979; Ludwig 2000, 2001; Antonín et al. 2011).

#### ﻿ Gerronema brunneosquamulosum

Taxon classificationFungiAgaricalesPorotheleaceae

Q.Na & Y.P.Ge sp. nov.

6C4B7306-B481-5E28-8B7C-C8AA10E358D4

849408

Figs 5 , 6 , 7

##### Diagnosis.

Differs from *G.zhujian* in having a fuscous stipe densely covered with deep-brown pubescence or scales and by the presence of large basidiospores.

**Figure 5.**
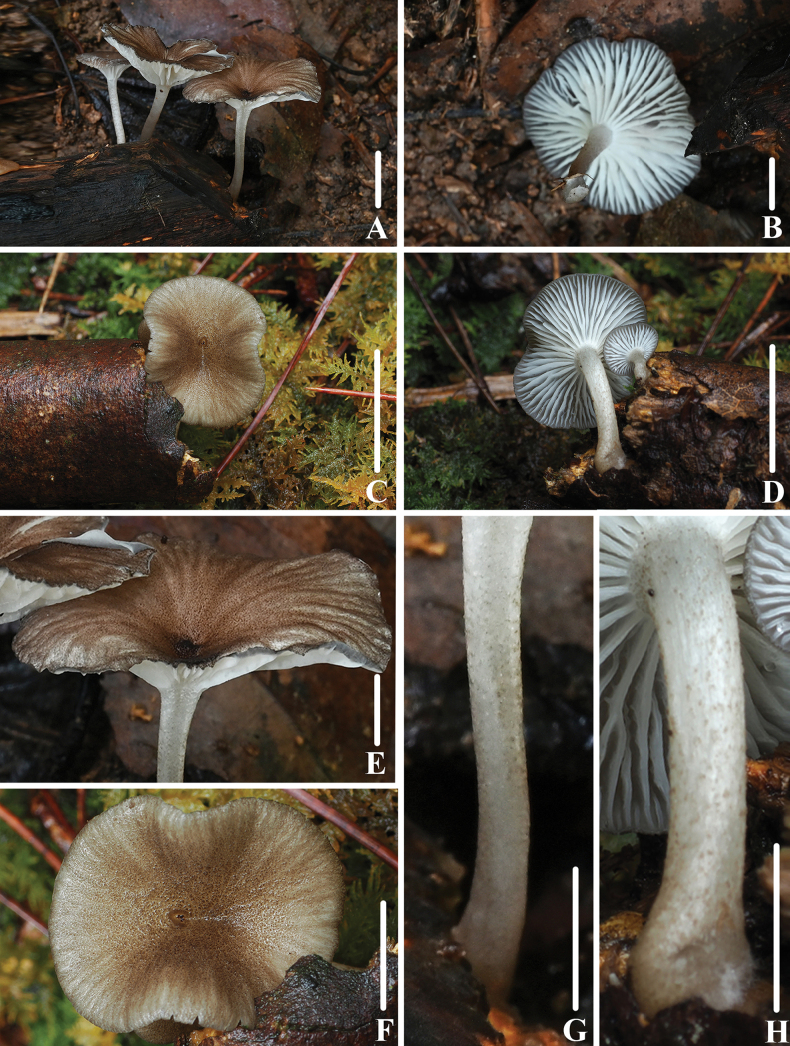
Basidiomata of *Gerronemabrunneosquamulosum***A, B***FFAAS1032*, holotype **C, D** collection *FFAAS1033***E, F** pileus with granules, fur or scales **G, H** stipe covered with dark brown scales. Scale bars: 10 mm (**A–E**); 5 mm (**F–H**).

##### Holotype.

China. Zhejiang Province: Baiyun National Forest Park, Liandu District, Lishui City, 2 Aug 2021, Qin Na, Yupeng Ge, and Hui Zeng, *FFAAS1032* (collection number MY0481).

**Figure 6.**
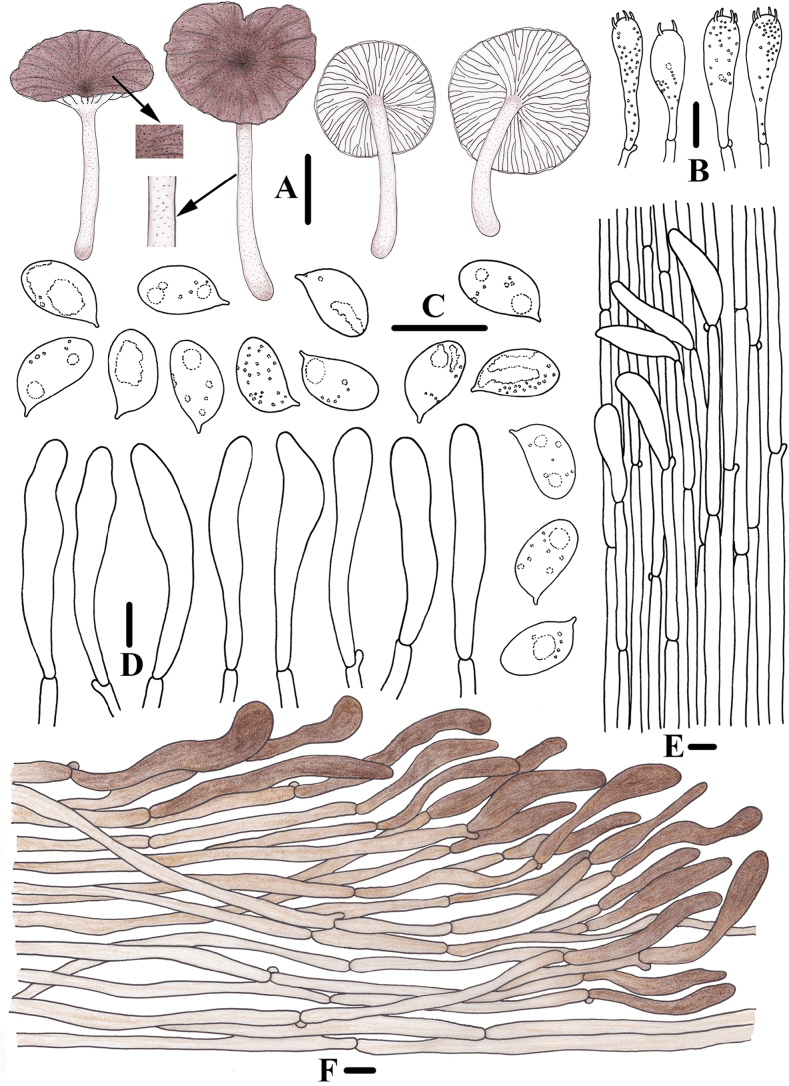
Morphological features of *Gerronemabrunneosquamulosum* (*FFAAS1032*, holotype) **A** basidiomata **B** basidia **C** basidiospores **D** cheilocystidia **E** caulocystidia **F** pileipellis. Scale bars: 10 mm (**A**); 10 μm (**B–F**).

##### Etymology.

Name refers to the pileus and stipe covered with dark-brown scales.

**Figure 7.**
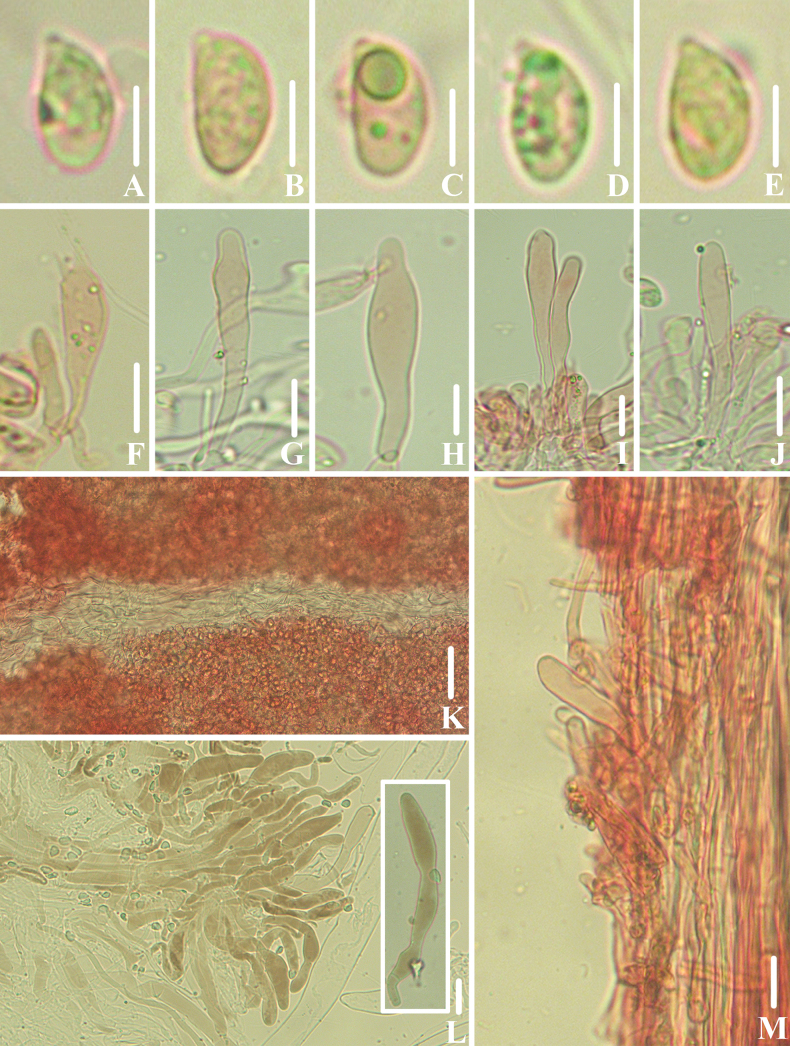
Microscopic features of *Gerronemabrunneosquamulosum* (*FFAAS1032*, holotype) **A–E** basidiospores **F** basidia **G–J** cheilocystidia **K** lamellar trama **L** pileipellis and pileocystidia **M** caulocystidia. Scale bars: 5 μm (**A–E**); 10 μm (structures **A**–**K, M** were stained with 1% Congo Red aqueous solution and **L** in 5% KOH aqueous solution before photographing).

##### Description.

Pileus 4.5–42.0 mm in diam., applanate and centrally depressed, subumbilicate to umbilicate when young, concave to deeply infundibulate with age, pellucid-striate or sulcate, always ± distinctly radially striped with darkened lines, Buffy Brown (XL17′′′k) at the centre, Olive Buff (XL21′′′d) in margin when young, Olive Brown (XL17′′′k), Clove Brown (XL17′′′m), Light Greyish-Olive (XLVI21′′′′b) in margin with age, densely covered with tiny, Warm Blackish-Brown (XXXIX1′′′m) granules, pubescence or scales, slightly sparse with age, dry, lustreless, with a slightly involuted margin. Context white, thin, tough. Lamellae narrowly adnexed to subdecurrent, moderately broad, pure white, edges concolorous with the sides. Stipe 6.0–32.0 × 1.5–2.0 mm, central, cylindrical, almost equal above, white, densely covered with Warm Blackish-Brown (XXXIX1′′′m) scales, hollow, base Light Seal Brown (XXXIX9′′′m), slightly swollen with tiny, inconspicuous fine white hairs. Odourless. Taste mild.

Basidiospores [60/3/2] (9.0) 9.2–10.0–11.2 (12.9) × (4.9) 5.2–5.8–6.6 (7.2) μm [Q = 1.54–1.91, Q = *1.73* ± 0.097] [holotype [40/2/1] (9.0) 9.2–10.2–11.2 (12.9) × (5.3) 5.5–5.9–6.5 (7.2) μm, Q = 1.54–1.90, Q = *1.71* ± 0.086], ellipsoid to narrowly ellipsoid, hyaline, guttulate, thin-walled, inamyloid. Basidia 22–39 × 7–9 μm, hyaline, clavate, 2- or 4-spored, sterigmata 2.3–6.0 × 0.8–2.2 μm. Cheilocystidia 23–59 × 6–9 μm, subfusiform, clavate, apex usually swollen, hyaline. Pleurocystidia absent. Lamellar trama subregular; hyphae 2–7 μm wide, thin-walled, hyaline, inamyloid. Pileus trama subregular, sarcodimitic. Pileipellis hyphae 3–7 μm wide, a cutis, light yellow (2B2); terminal elements clavate or utriform with rounded apex, 53–95 × 7–16 μm, Dark Citrine (IV21m), Olive Brown (XL17′′′k) to Clove Brown (XL17′′′k) pigmented; true pileocystidia absent. Hyphae of the stipitipellis 5–11 μm wide, hyaline, smooth; caulocystidia long cylindrical, sometimes with rounded apex, 40–76 × 5–12 μm, hyaline, thin-walled. All tissues non-reactive in iodine. Clamps present in all tissues.

##### Habit and habitat.

Solitary to scattered on rotten wood, branches and twigs in *Acer*, *Ginkgo*, *Liriodendron*, *Picea* and *Tsuga*.

##### Known distribution.

Fujian Province, Zhejiang Province, China.

##### Additional material examined.

China. Fujian Province: Wuyi Mountain, Nanping City, 13 Aug 2021, Qin Na, Yupeng Ge, Junqing Yan, Hui Zeng, and Zewei Liu, *FFAAS1033* (collection number MY0571).

##### Notes.

*Gerronemabrunneosquamulosum* is unique amongst members of *Gerronema* on account of its fuscous pileus and stipe with dark-brown to blackish-brown pubescence or scales, larger basidiospores and a dark-pigmented pileipellis. *Gerronemazhujian*, reported from Anhui and Fujian Provinces in China, is the most closely allied congener of *G.zhujian* on the basis of the brown colouration of the umbilicus of its pileus, its whitish stipe and similarly-shaped cheilocystidia and terminal elements of the pileipellis (Na et al. 2022a). This taxon differs from *G.brunneosquamulosum* in having a pruinose white stipe, subdecurrent to decurrent lamellae and possessing smaller basidiospores (Na et al. 2022a). Two species of *Omphalina* Quél., characterised by dark pigments in the pileus, have been described from Argentina–*Omphalinadepauperata* (Singer) Raithelh. and *O.subpallida* (Singer) Raithelh., formerly named *Gerronemasubpallidum* Singer and *G.depauperatum* Singer, respectively. These two species most closely resemble *G.brunneosquamulosum*, but differ in having an unornamented stipe, ellipsoid basidiospores and no cheilocystidia (Singer 1970). Other species of *Gerronema*, such as *G.nemorale* and *G.strombodes*, are well characterised with a distinctly yellow, yellowish-orange, olive-yellow to yellowish-brown pileus and their micromorphological features are also different (Singer 1970; Antonín et al. 2008; Latha et al. 2018). Species of *Trogia*, especially *Trogiafulvochracea* Corner (p.31) and *Trogiamycenoides* (p.53) Corner, share some similarities with the new taxon (Corner 1991). *Trogiafulvochracea*, however, has a fulvous or cinnamon-ochraceous pileus, a smooth white stipe and smaller basidiospores (7–9.5 × 4.5–6.0 μm). *Trogiamycenoides* differs in having a smaller pileus (5–30 mm in diam.), ellipsoid basidiospores and clavate to subglobose cheilocystidia; in addition, true pileocystidia are present, but are soon evanescent (Corner 1991).

#### ﻿ Leucoinocybe subglobispora

Taxon classificationFungiAgaricalesPorotheleaceae

Q.Na & Y.P.Ge sp. nov.

0C3BD52F-4813-5C08-9B64-12B32B5C8DCC

849409

Figs 8 , 9 , 10

##### Diagnosis.

Pileus dark brown. Basidiospores subglobose to broadly ellipsoid. Pileocystidia and caulocystidia thick-walled. Differs from *L.lishuiensis* in having broader basidiospores.

**Figure 8.**
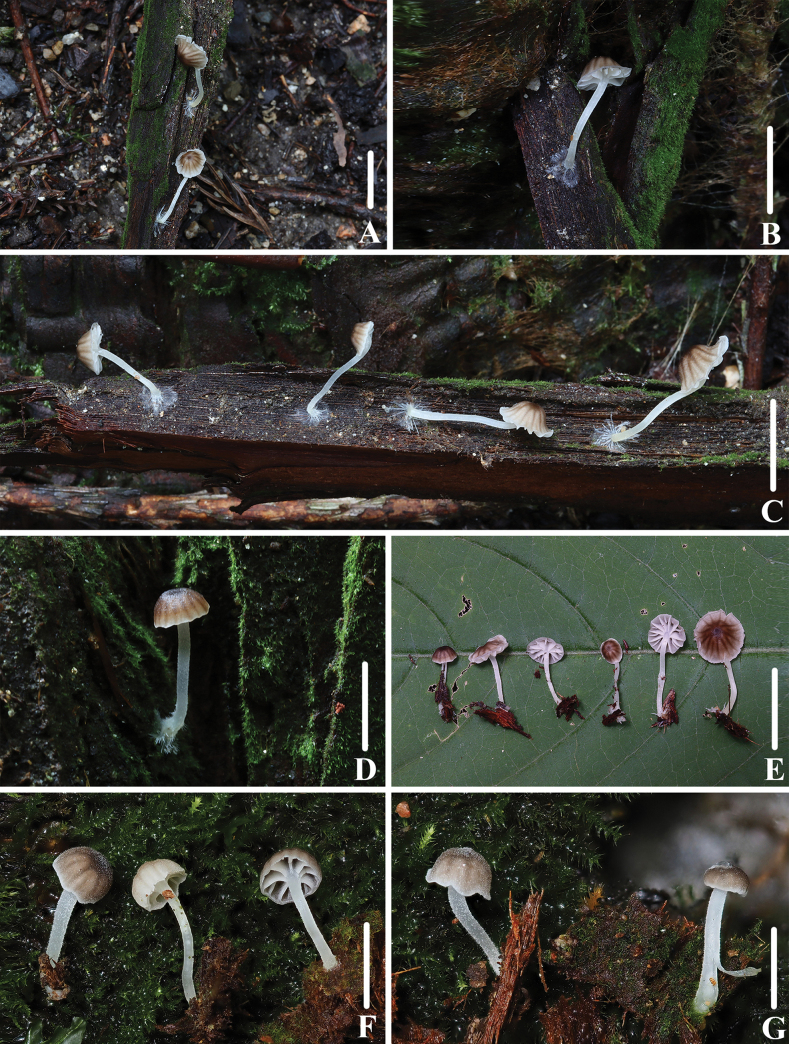
Basidiomata of *Leucoinocybesubglobispora***A–E** collection *FFAAS1034*, holotype **F–G** collection *FFAAS1035*. Scale bars: 10 mm (**A–G**).

##### Holotype.

China. Zhejiang Province: Tianmu Mountain, Hangzhou City, 1 Aug 2021, Qin Na, Yupeng Ge, Zewei Liu and Yulan Sun, *FFAAS1034* (collection number MY0444).

**Figure 9.**
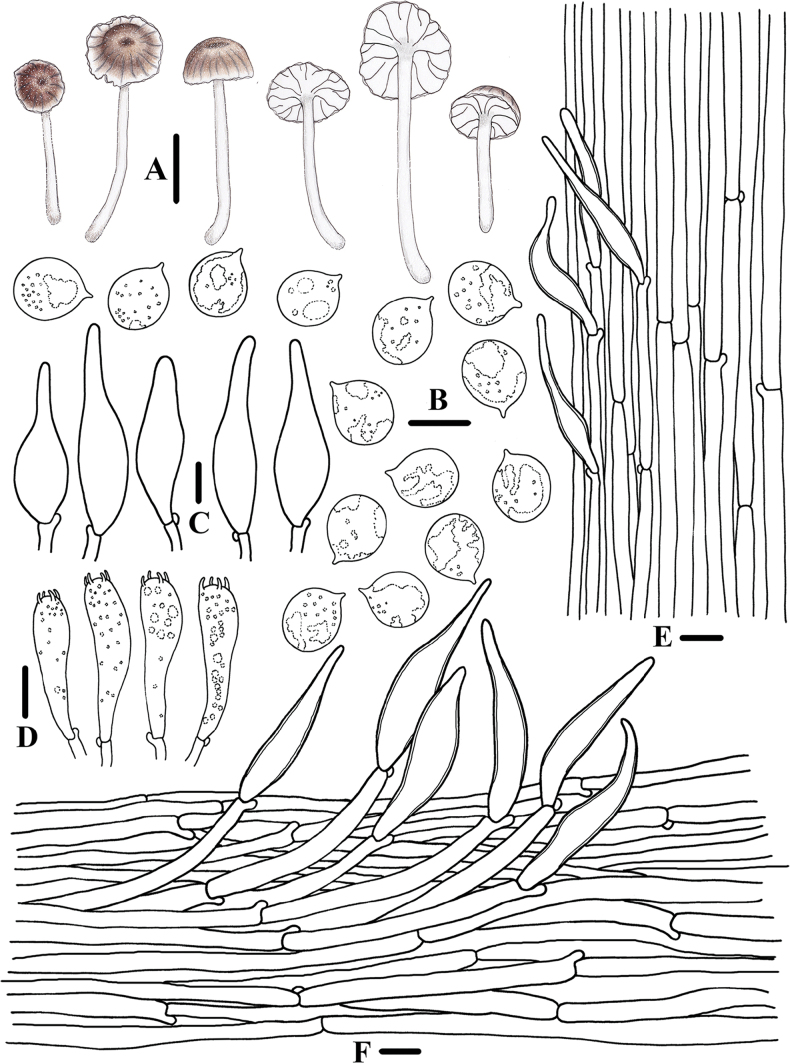
Morphological features of *Leucoinocybesubglobispora* (*FFAAS1034*, holotype) **A** basidiomata **B** basidiospores **C** cheilocystidia **D** basidia **E** caulocystidia **F** pileocystidia. Scale bars: 5 mm (**A**); 10 μm (**B–F**).

##### Etymology.

Name refers to the subglobose to broadly ellipsoid basidiospores.

**Figure 10.**
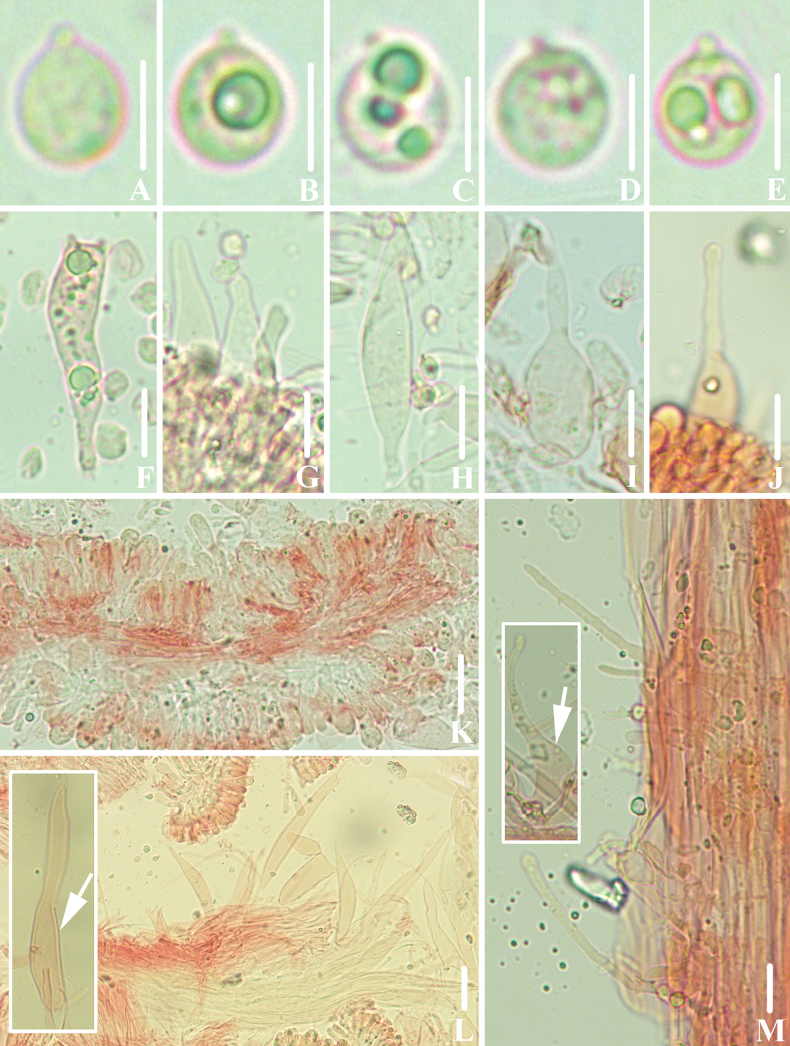
Microscopic features of *Leucoinocybesubglobispora* (*FFAAS1034*, holotype) **A–E** basidiospores **F** basidia **G**–**J** cheilocystidia **K** lamellar trama **L** pileipellis and pileocystidia **M** caulocystidia. Scale bars: 5 μm (**A–E**); 10 μm (**F–M**). Structures were stained with 1% Congo Red aqueous solution before photographing.

##### Description.

Pileus 2.5–8.0 mm in diameter, hemispherical or campanulate when young, becoming campanulate with age, umbilicate at the centre, sulcate, finely granulose all over, Dark Livid Brown (XXXIX1′′′k), Benzo Brown (XLVI13′′′′i) to Fuscous (XLVI13′′′′k) at the centre, Pale Smoke Grey (XLVI21′′′′f) in the margin, uplifted or recurved at the margin and sometimes rimose in age, dry. Context white, thin, fragile. Lamellae adnexed to slightly subdecurrent, white, with 1–2 tiers of lamellulae, edges concolorous with the face. Stipe 9.5–14.0 × 1.0–1.5 mm, equal or slightly broadened at the base, hollow, fragile, white, sometimes inconspicuous Pale Olive-Buff (XL21′′′d) at the base, densely pruinose, but sparsely with age, base covered with small white fibrils. Odour and taste indistinctive.

Basidiospores (60/3/2) (5.6) 5.8–6.4–7.1 (7.5) × (4.8) 5.0–5.6–6.5 (6.8) μm [Q = 1.06–1.27, Q = *1.16* ± 0.054] [holotype (40/2/1) (5.7) 5.9–6.5–7.2 (7.5) × (4.9) 5.0–5.5–6.5 (6.8) μm, Q = 1.07–1.27, Q = *1.18* ± 0.052], subglobose to broadly ellipsoid, hyaline in 5% KOH, smooth, thin-walled, guttulate, amyloid. Basidia 28–37 × 7–9 μm, 4-spored, clavate, sterigmata 1.4–2.7 × 0.8–1.7 μm. Cheilocystidia 28–62 × 9–15 μm, distinct, flexuose, narrowly utriform, fusoid or lageniform, subcapitate, thin-walled, hyaline. Pleurocystidia absent. Lamellae trama subregular; hyphae 2–6 μm wide, thin-walled, hyaline, amyloid. Pileipellis hyphae 2–8 μm wide, smooth; pileocystidia 62–116 × 10–19 μm, lageniform, subulate, apically obtuse, distinctly 0.8–1.8 μm thick-walled, with a thin-walled base, hyaline, smooth. Stipitipellis a cutis made up of 3–9 μm wide hyphae, smooth, thin-walled; caulocystidia 34–62 × 5–10 μm, subulate, fusoid, lageniform, sometimes clavate, always thick-walled in the middle part and with a thin-walled base, smooth, transparent. Clamps present in all tissues.

##### Habit and habitat.

Solitary or scattered on rotten wood or branches in *Acer*, *Armeniaca*, *Cercidiphyllum*, *Emmenopterys* and *Picea* mixed forests.

##### Known distribution.

Zhejiang Province, China.

##### Additional material examined.

China. Zhejiang Province: Baiyun National Forest Park, Liandu District, Lishui City, 2 Aug 2021, Qin Na, Yupeng Ge, Zewei Liu, Yaping Hu and Hui Ding, *FFAAS1035* (collection number MY0475).

##### Notes.

*Leucoinocybesubglobispora* is considered to be a distinct species of *Leucoinocybe* on account of its subdecurrent lamellae, subglobose to broadly ellipsoid basidiospores, thick-walled pileocystidia and caulocystidia and saprophytic habitat. *Leucoinocybelenta*, the type species of *Leucoinocybe*, also has a white stipe and lamellae, similarly-shaped cheilocystidia and thick-walled pileocystidia, but differs from the new species by the presence of a reddish-brown pileus with pinkish shades or pale pinkish-beige at the centre that fades to white towards the margin, larger basidiomata and ellipsoid basidiospores [(5.3)6.0–7.3(7.9) × (3.8)4.0–4.5(5.1) μm] (Gröger 2006; Eyssartier and Roux 2011; Antonín et al. 2019; Kaygusuz et al. 2020). *Leucoinocybetaniae* (= *Clitocybulaflavoaurantia*) resembles *L.subglobispora* in having a brown pileus, white and decurrent lamellae and a white stipe with a brownish base, but differs in possessing the following features: a reddish-yellow pileus when old, larger and broadly amygdaliform spores (6.2–7.8 × 4.8–7.0 μm) and thin-walled pileocystidia and caulocystidia (Vila 2002; Contu 2003; Malysheva and Morozova 2011; Antonín et al. 2019). *Leucoinocybesulcata*, recently described as a new taxon from India, is easily distinguished from the new species by the presence of greyish-orange to brown basidiomata, a larger pileus (13–52 mm in diam.), broadly ellipsoid to subamygdaliform basidiospores (5.0–6.5 × 4.0–5.5 μm; Q = 1.1–1.5) and thin-walled caulocystidia and the absence of pileocystidia (Latha et al. 2015). *Leucoinocybelishuiensis*, reported as a new species from south-eastern China in our previous study, can be easily mistaken for *L.subglobispora* on account of having an identical habit and habitat, a small, pure-brown pileus, slightly decurrent lamellae, similarly-shaped cheilocystidia and thick-walled pileocystidia and caulocystidia; however, the narrowly ellipsoid basidiospores and smaller pileocystidia of *L.lishuiensis* can be used to distinguish this species from *L.subglobispora* (Na et al. 2021). Another new combination of *Leucoinocybe*, *L.auricoma* (Har. Takah.) Matheny, originally named *Mycenaauricoma* Har. Takah., is also comparable to the present species in having thick-walled pileocystidia and caulocystidia; however, *L.auricoma* has a yellowish-orange flocculent pileus and stipe, ovoid-ellipsoid to ellipsoid basidiospores (5–7 × 3–4 μm) and pileocystidia and caulocystidia with yellow contents (Takahashi 1999; Matheny et al. 2020).

#### ﻿ Marasmiellomycena albodescendens

Taxon classificationFungiAgaricalesPorotheleaceae

(J.A. Cooper) Q.Na & Y.P.Ge comb. nov.

42B4EF77-26AB-54F6-931B-E6DCA0579A8F

851718

##### Basionym.

*Porotheleumalbodescendens* J.A. Cooper, in Consiglio, Vizzini, Cooper, Marchetti, Angelini, Brugaletta & Setti, Riv. Micol. 64(2): 117, 2022.

##### Type specimen.

***Holotype***: New Zealand: North Island, Taupo, Tauhara Centre, 15 May 2011, PDD 96321.

##### Selected description.

Consiglio et al. (2022).

##### Distribution.

New Zealand.

##### Notes.

*Marasmiellomycenaalbodescendens* has marasmielloid basidiomes, a pure-white pileus, relatively large spores, no hymenial cystidia and abundant, thick-walled pileocystidia and caulocystidia with yellowish contents. Unlike other species of *Marasmiellomycena* possessing a yellow, reddish-brown or yellowish-brown pileus, *M.albodescendens* can be easily recognised by its white pileus. The pileus of *Marasmiellomycenaalbodescendens* is macromorphologically more similar to some species of *Marasmiellus* Murrill (Stevenson 1964); however, its micromorphological characteristics place this species in *Marasmiellomycena*, consistent with the results of our phylogenetic analysis (Fig. 1). *Marasmiellomycenaalbodescendens* has been infrequently collected in New Zealand, but is probably common and widespread and grows on small, dead, fallen branches and twigs in indigenous scrub and broad-leaf forests in late summer and autumn (Consiglio et al. 2022).

#### ﻿ Marasmiellomycena tomentosa

Taxon classificationFungiAgaricalesPorotheleaceae

Q.Na & Y.P.Ge sp. nov.

75656C5C-F842-5871-BDDC-ACDA1742F6A7

851717

**Figure 11.**
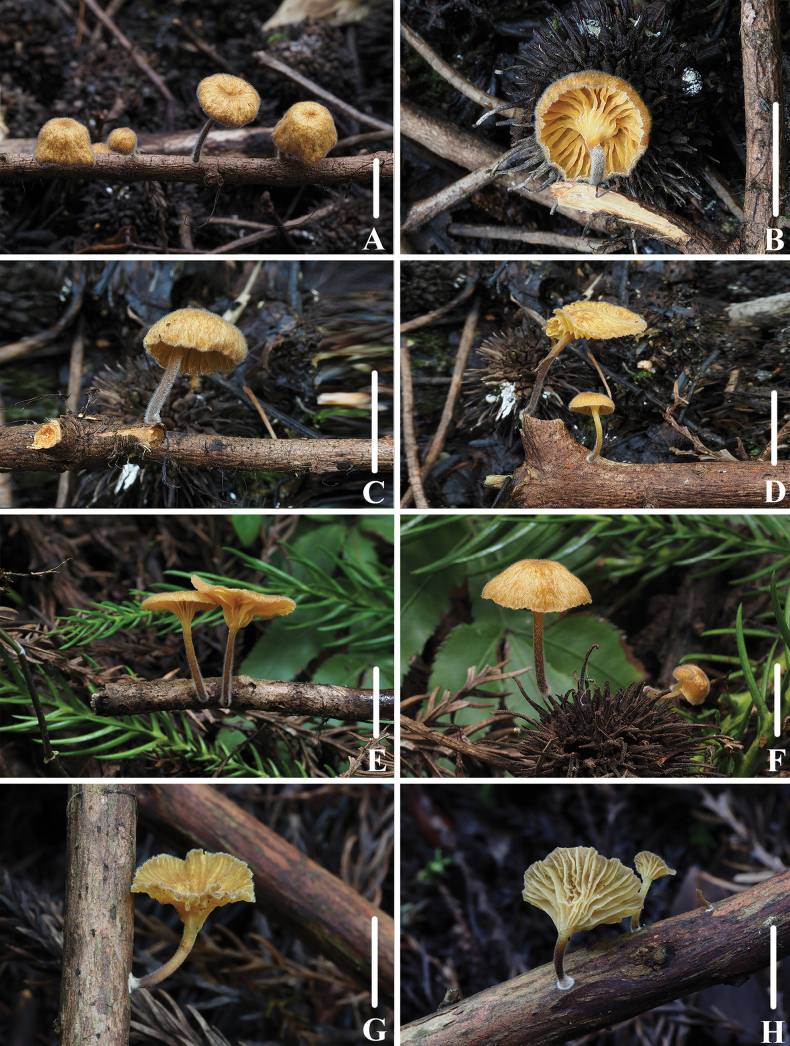
Basidiomata of *Marasmiellomycenatomentosa***A–D** collection *FFAAS1036*, holotype **E, F** collection *FFAAS1037***G, H** collection *FFAAS1038*. Scale bars: 10 mm (**A–H**).

**Figure 12.**
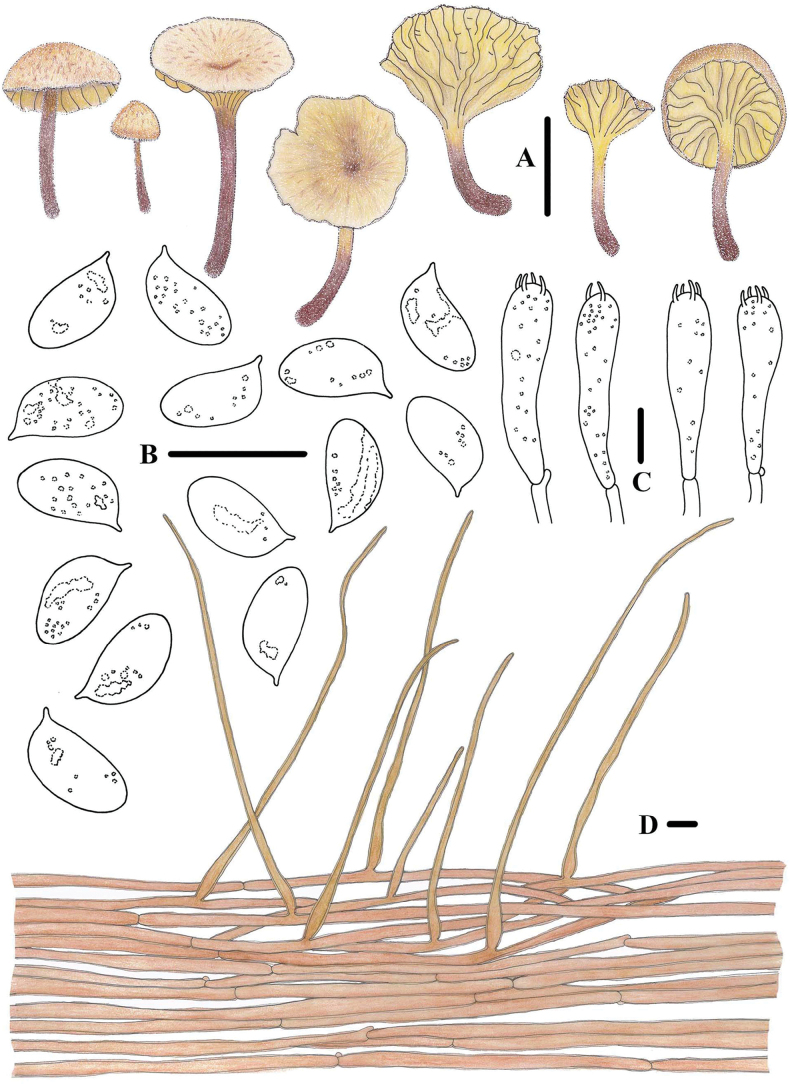
Morphological features of *Marasmiellomycenatomentosa* (*FFAAS1036*, holotype) **A** basidiomata **B** basidiospores **C** basidia **D** pileipellis and pileocystidia. Scale bars: 10 mm (**A**); 10 μm (**B–D**).

**Figure 13.**
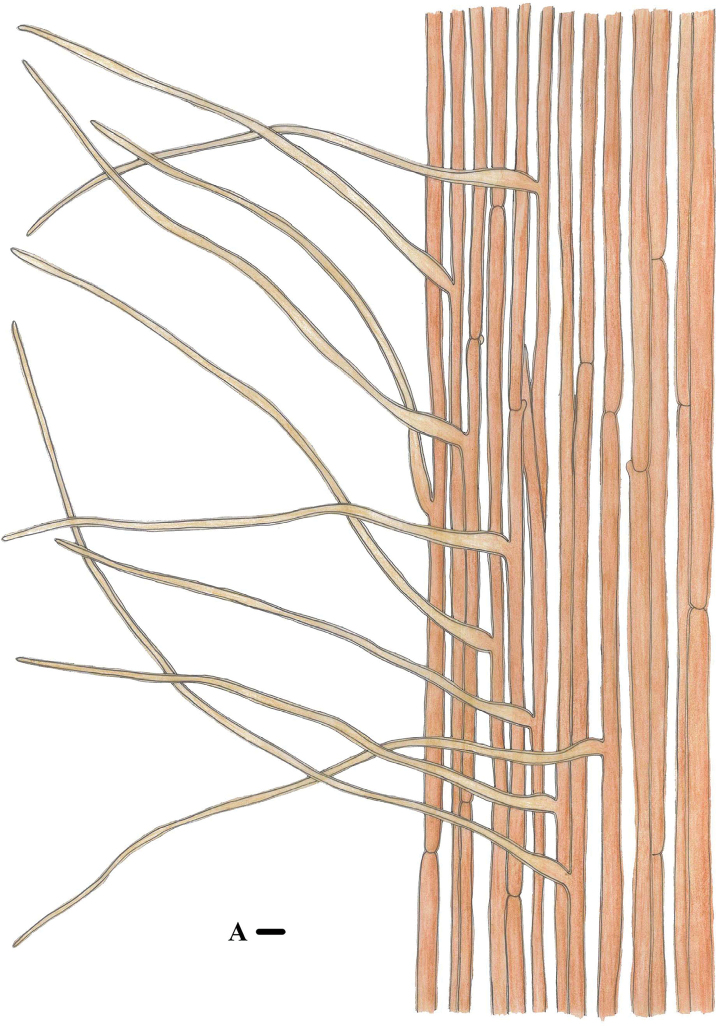
Morphological features of *Marasmiellomycenatomentosa* (*FFAAS1036*, holotype) **A** stipitipellis and caulocystidia. Scale bars: 10 μm (**A**).

**Figure 14.**
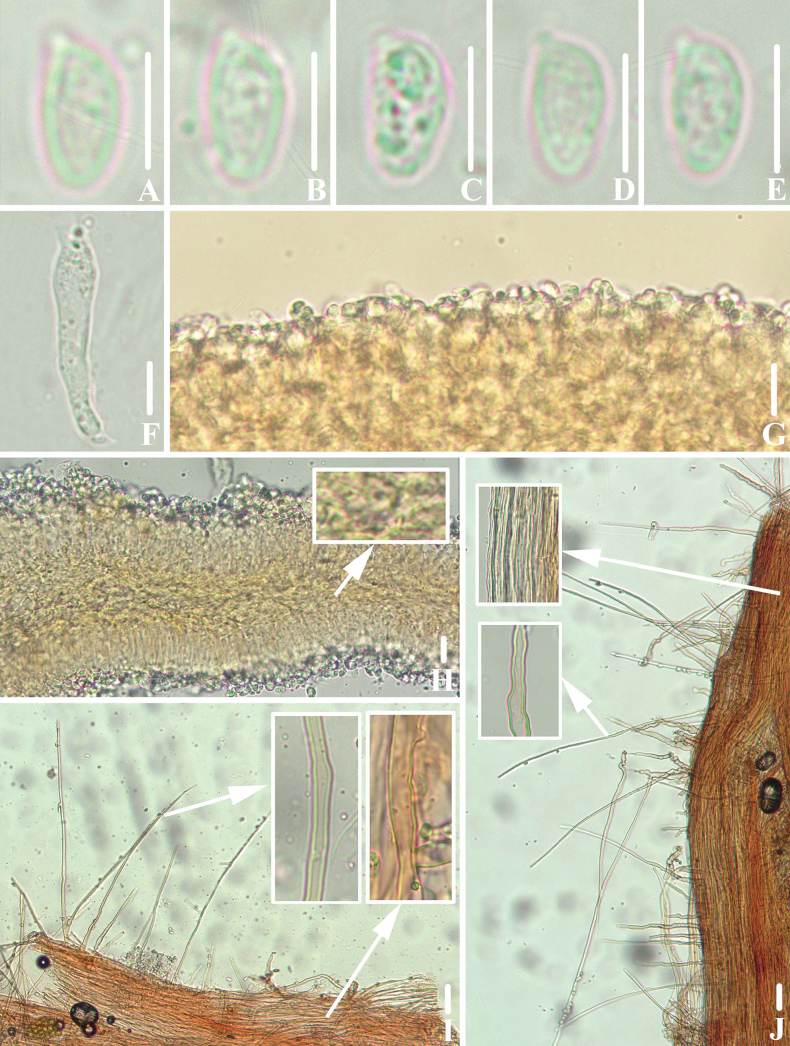
Microscopic features of *Marasmiellomycenatomentosa* (*FFAAS1036*, holotype) **A–E** basidiospores **F** basidia **G**–**J** lamellae margin. Scale bars: 5 μm (**A–E**); 10 μm (**F–J**). Structures were stained in 5% KOH aqueous solution before photographing.

Figs 11 , 12 , 13 , 14

##### Diagnosis.

Pileus and stipe distinctly tomentose. Pileus dark brown, subsquamulose. Basidiospores narrowly ellipsoid, slightly amyloid. Hymenial cystidia absent. Pileipellis and stipitipellis sarcodimitic, hyphae thick-walled with yellowish-brown pigments. Pileocystidia and caulocystidia thick-walled with yellow contents. Differs from *M.pseudoomphaliiformis* by possessing a distinctly tomentose, dark-brown subsquamulose pileus, narrowly ellipsoid basidiospores and absence of cheilocystidia.

##### Holotype.

China. Zhejiang Province: Tianmu Mountain, Hangzhou City, 30 Jul 2021, Qin Na, Zewei Liu, Yulan Sun and Yupeng Ge, *FFAAS1036* (collection number MY0421).

##### Etymology.

Name refers to the tomentose to subsquamulose pileus.

##### Description.

Pileus 0.5–18.5 mm in diameter, at first convex or campanulate, soon expanding to plano-convex, always depressed to umbilicate at the centre, surface dry, densely covered with minute white (LIII) pubescence, tomentose all over, subsquamulose, ground colour Verona Brown (XXIX13′′k) to Warm Sepia (XXIX13′′m), Mustard Yellow (XVI19′b), Old Gold (XVI19′i) to Buffy Citrine (XVI19′k), Saccardo’s Olive (XVI19′m) at the centre, fading to Wax Yellow (XLVI21′′′′f) when old, margin slightly sulcate, uplifted or recurved in age. Context thin, Primrose Yellow (XXX23′′d). Lamellae decurrent to subdecurrent, Wax Yellow (XLVI21′′′′f), Mustard Yellow (XVI19′b), with 1–2 tiers of lamellulae, edges concolorous with the face, slightly fimbriate edge. Stipe 7.5–21.0 × 1.0–1.6 mm, central, terete, curved, equal or slightly broadened at the base, hollow or stuffed, dry, Mustard Yellow (XVI19′b) in the upper part, Saccardo’s Olive (XVI19′m), Benzo Brown (XLVI13′′′′i), Fuscous (XLVI13′′′′m), Deep Greyish-Olive (XLVI21′′′′b) towards the base, densely and minutely silky-fibrillose and white (LIII) pruinose-floccose to tomentose throughout, base covered with white mycelium. Odour indistinct to fungoid, taste mild.

Basidiospores (80/4/3) (6.8) 7.2–7.6–8.2 (8.4) × (3.7) 3.9–4.1–4.5 (4.6) μm [Q = 1.75–1.98, Q = *1.83* ± 0.052] [holotype (40/2/1) (6.8) 7.2–7.7–8.4 × 3.9–4.2–4.6 μm, Q = 1.75–1.98, Q = *1.82* ± 0.050], narrowly ellipsoid, hyaline in 5% KOH, smooth, thin-walled, multiguttulate, slightly amyloid. Basidia 20–35 × 5–8 μm, 2- or 4-spored, clavate, sterigmata 2.2–4.8 × 0.6–1.6 μm. Hymenial cystidia absent. Lamellar trama subregular; hyphae 3–10 μm wide, with 0.5–1.0 µm thick-walled, light yellow, dextrinoid. Pileipellis hyphae 3–8 μm wide, sarcodimitic, cutis, smooth, 0.4–1.0 μm thick-walled, with intracellular yellowish-brown pigment; pileocystidia 38–223 × 5–12 μm, in clusters, narrowly subulate or narrowly lageniform to fusiform with very long and tapering neck, distinctly 0.6–1.5 μm thick-walled, yellow, smooth. Stipitipellis made up of cylindrical, 4–9 µm wide hyphae, sarcodimitic, smooth, 0.5–1.0 μm thick-walled, with intracellular brownish-orange pigment; caulocystidia 45–327 × 5–9 μm, similar to the pileocystidia, but usually longer, 0.5–1.3 μm thick-walled, smooth, with intracellular yellowish pigment. Clamps present in all tissues.

##### Habit and habitat.

Solitary or scattered on rotten branches, twigs and wood debris in *Acer*, *Armeniaca*, *Cercidiphyllum*, *Emmenopterys* and *Picea* mixed forests.

##### Known distribution.

Zhejiang Province, China.

##### Additional material examined.

China. Zhejiang Province: Tianmu Mountain, Hangzhou City, 30 Jul 2021, Qin Na, Zewei Liu, Yulan Sun and Yupeng Ge, *FFAAS1037* (collection number MY0422); Zhejiang Province: Tianmu Mountain, Hangzhou City, 1 Aug 2021, Qin Na, Zewei Liu, Yulan Sun and Yupeng Ge, *FFAAS1038* (collection number MY0443).

##### Notes.

*Marasmiellomycenatomentosa* is a rare thermophilous species reported from south-eastern areas of China from July to August on rotten branches, twigs and woody debris of deciduous and coniferous trees (*Acer*, *Armeniaca*, *Cercidiphyllum*, *Emmenopterys* and *Picea*). The most distinctive characteristics of this species are a tomentose, brown subsquamulose pileus, a tomentose stipe, narrowly ellipsoid and slightly amyloid basidiospores, the absence of hymenial cystidia and thick-walled pileipellis, stipitipellis, pileocystidia and caulocystidia with yellow or brownish-orange contents. Species morphologically most closely allied to *Marasmiellomycenatomentosa* include *M.omphaliiforme*, *M.pseudoomphaliiformis* and *M.albodescendens*. *Marasmiellomycenapseudoomphaliiformis* resembles *M.tomentosa* by the presence of a pale beige to brown pileus with finely tomentose to pubescent pileus, but differs in having white to cream-white or beige lamellae rather than yellow, ellipsoid to ellipsoid-fusiform basidiospores [(6.5–)7.0–9.0(–9.5) × 4.0–5.5 µm] and clavate, fusiform to lageniform cheilocystidia (Senanayake et al. 2023). *Marasmiellomycenaomphaliiforme* is considered to be a closely-related taxon with evident affinities to *M.tomentosa*–not only regarding its phylogenetic placement, but also in terms of morphological features (Kühner and Romagnesi 1954; Antonín and Noordeloos 1993, 1997; Consiglio et al. 2022; Senanayake et al. 2023). The two species resemble one another in having a similarly-coloured pileus and stipe, similarly-shaped basidiospores, pileocystidia and caulocystidia and a yellowish-pigmented pileipellis and stipitipellis; however, the minutely pubescent, granulose to subsquamulose pileus, as well as the relative abundance of cheilocystidia, appear to be variable characters in *M.omphaliiforme* in contrast to the new species (Kühner and Romagnesi 1954; Antonín and Noordeloos 1993, 1997; Consiglio et al. 2022). According to the description of Consiglio et al. (2022), *Marasmiellomycenaalbodescendens* from New Zealand has a pure-white pileus, a thin-walled pileipellis and larger basidiospores (9.6 ± 0.7 µm × 5.2 ± 0.4 µm).

#### ﻿ Pulverulina flavoalba

Taxon classificationFungiAgaricalesPorotheleaceae

Q.Na & Y.P.Ge sp. nov.

98572BA2-7764-5EFC-9ACF-C3272F864790

849410

Figs 15 , 16 , 17

##### Diagnosis.

Pileus white to light orange yellow. Basidiospores cylindrical. Hymenial cystidia absent. Lamellar trama, pileipellis and stipitipellis hyphae thin-walled. Differs from *Pu.ulmicola* in having larger and longer basidiospores and possessing thin-walled lamellar trama, pileipellis and stipitipellis hyphae.

**Figure 15.**
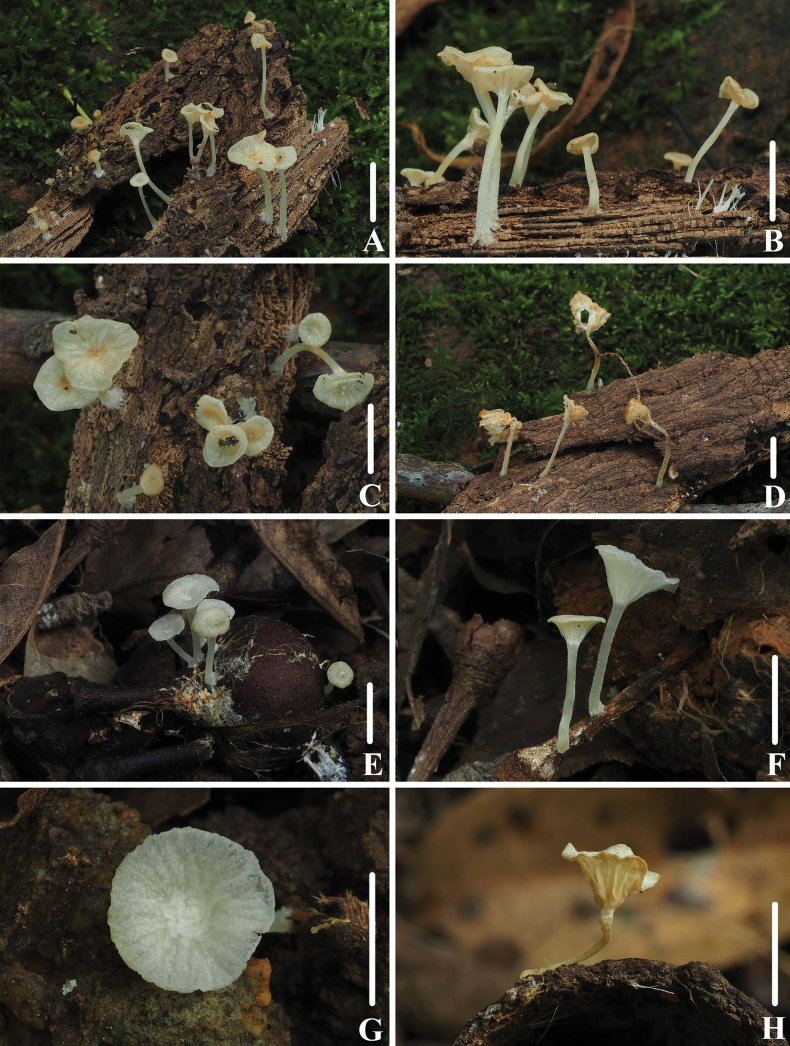
Basidiomata of *Pulverulinaflavoalba***A–D** collection *FFAAS1039*, holotype **E–H** collection *FFAAS1040*. Scale bars: 5 mm (**A–H**).

##### Holotype.

China. Guangxi Zhuang Autonomous Region: Liangfengjiang National Forest Park, Nanning City, 13 Jul 2022, Yupeng Ge and Renxiu Wei, *FFAAS1039* (collection number MY0863).

**Figure 16.**
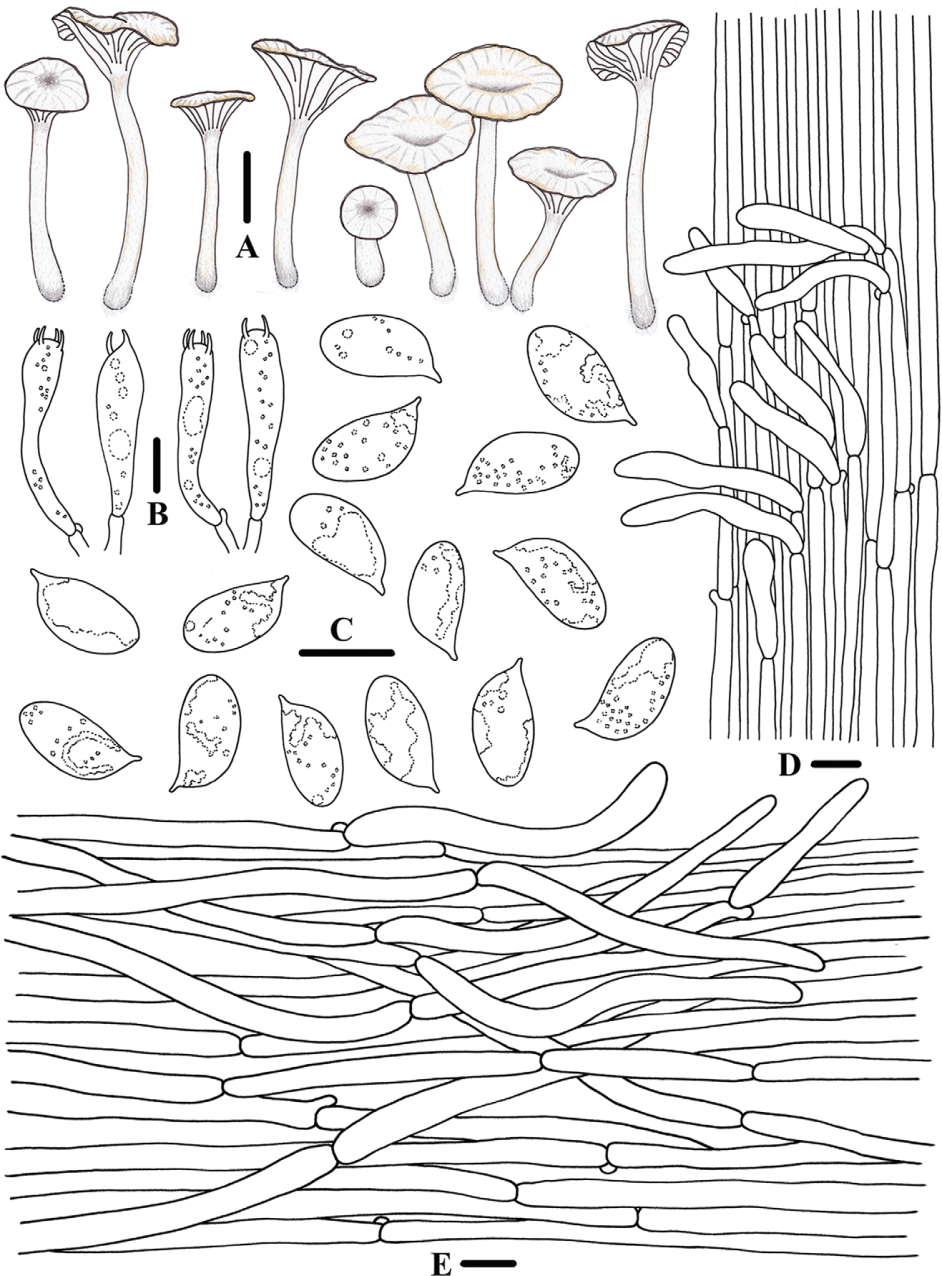
Morphological features of *Pulverulinaflavoalba* (*FFAAS1039*, holotype) **A** basidiomata **B** basidia **C** basidiospores **D** caulocystidia **E** pileipellis. Scale bars: 2 mm (**A**); 10 μm (**B, D, E)**; 5 μm (**C**).

##### Etymology.

Name refers to the white to light-yellow pileus and stipe.

**Figure 17.**
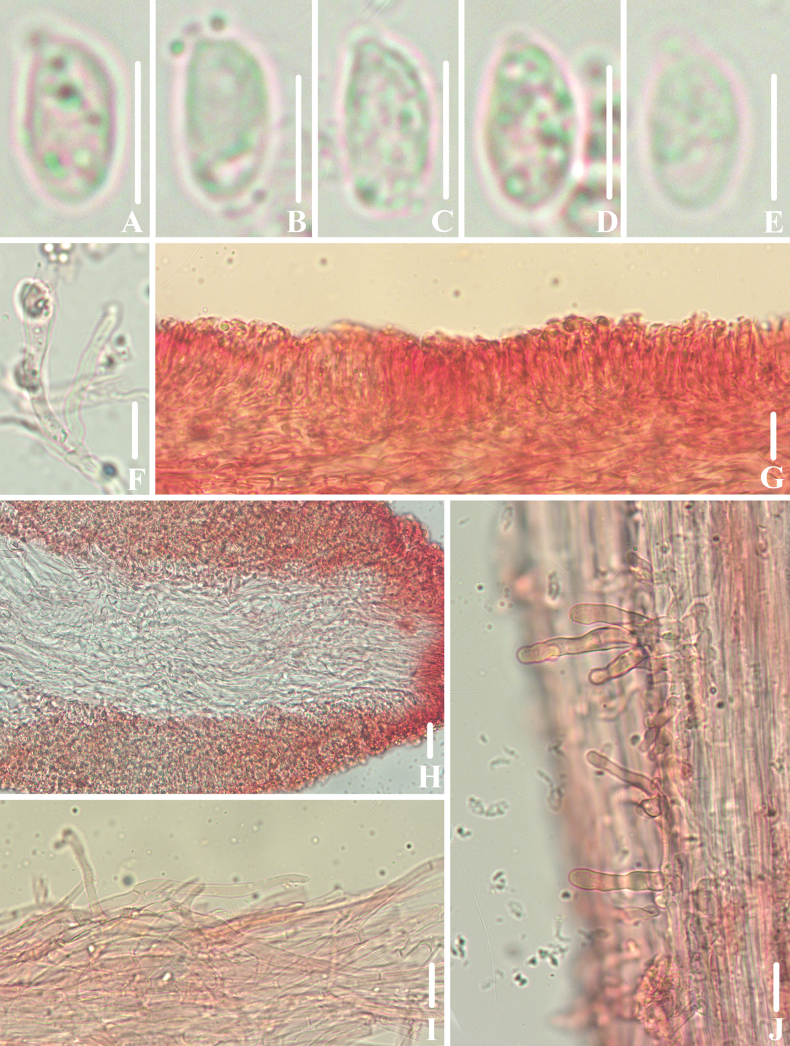
Microscopic features of *Pulverulinaflavoalba* (*FFAAS1039*, holotype) **A**–**E** basidiospores **F** basidia **G** lamellae margin **H** lamellar trama **I** pileipellis **J** caulocystidia. Scale bars: 5 μm (**A–E**); 10 μm (**F–J**). Structures **A–F** were stained in 5% KOH aqueous solution and **G–J** with 1% Congo Red aqueous solution before photographing.

##### Description.

Pileus 1.2–5.8 mm in diameter, arched or plano-convex with a slight depression at the centre when young, becoming more depressed with age; translucent striate, floccose or granulose, glabrescent when old, surface dull, dry; white (LIII) when young, aniline yellow (IV19i) or light orange-yellow (III17d) at the centre and in the margin with age, margin decurved. Context white, thin, not fragile. Lamellae decurrent, white, orange citrine (IV19k) tinged when old, with 1–2 tiers of lamellulae, edges even, medium-broad. Stipe 1.6–14.4 × 0.5–1.0 mm, terete or slightly broadened at the base, curved, dry, white, with a pruinose, pubescent or fibrillose surface, sparser with age, hollow, not fragile, white, sometimes aniline yellow (IV19i), light orange-yellow (III17d) in the middle and at the base; base covered with white mycelium. Odour absent, taste mild.

Basidiospores (60/3/2) (6.8) 7.0–7.9–8.8 (9.1) × (3.3) 3.7–4.1–4.4 (4.7) μm [Q = 1.81–2.19, Q = *1.93* ± 0.099] [holotype (40/2/1) (6.8) 7.0–7.8–8.9 (9.1) × (3.3) 3.7–4.1–4.4 (4.7) μm, Q = 1.77–2.19, Q = *1.92* ± 0.084], cylindrical, hyaline in 5% KOH, smooth, thin-walled, guttulate, inamyloid, with a small, but discernible apiculus. Basidia 21–30 × 4–6 μm, 2- or 4-spored, clavate, sterigmata 1.9–5.6 × 0.6–1.6 μm. Hymenial cystidia absent. Lamellar trama subregular to interwoven; hyphae 5–15 µm wide, hyaline, thin-walled. Pileipellis a cutis of cylindrical hyphae 3–7 µm wide, smooth; end cells often protruding, 35–105 × 3–12 μm, cylindrical, subfusiform, apically obtuse, thin-walled, hyaline, smooth. Stipitipellis hyphae 3–8 μm wide, smooth, thin-walled; caulocystidia 19–50 × 4–9 μm, clavate, subfusiform, thin-walled, smooth, transparent. Clamps present in all tissues.

##### Habit and habitat.

Scattered to gregarious on rotten wood, branches or fruits in mixed forests of *Acacia*, *Ficus*, *Ilex*, *Parashorea*, *Picea* and *Trachycarpus* etc.

##### Known distribution.

Guangxi Zhuang Autonomous Region, China.

##### Additional material examined.

China. Guangxi Zhuang Autonomous Region: Liangfengjiang National Forest Park, Nanning City, 13 Jul 2022, Yupeng Ge and Renxiu Wei, *FFAAS1040* (collection number MY0865).

##### Notes.

*Clitocybeulmicola* H.E. Bigelow was established by Bigelow in 1982 and published as a new combination, *Pulverulinaulmicola* (H.E. Bigelow) Matheny & K.W. Hughes (Matheny et al. 2020). The description of *Pulverulinaulmicola* modified from Bigelow (1982) includes observations based on recent American material (Matheny et al. 2020). As far as we know, only *Pulverulinaulmicola* has previously been included in the genus and has had morphological features described in detail (Bigelow 1982; Matheny et al. 2020). In appearance, *Pulverulinaulmicola* is a small, whitish, marasmioid fungus, with small basidiomata, distant decurrent lamellae, a tough texture, interwoven gill trama, long cylindrical caulocystidia and short, ellipsoid, smooth basidiospores and occurs on the bark of living *Ulmus* and *Quercus* trees. Our collections of *Pulverulinaflavoalba* from the Guangxi Zhuang Autonomous Region represent a taxon that is distinct from *Pulverulinaulmicola*, as compared to the macroscopic and microscopic characters described by Matheny et al. (2020). *Pulverulinaulmicola* differs from *P.flavoalba* in having a white or whitish to very pale brown or faintly greyish pileus, broadly ellipsoid to ovoid basidiospores and lamellar trama, pileipellis and stipitipellis hyphae with thickened walls (Matheny et al. 2020). The *Pulverulina* genus comprises two additional species besides *Pulverulinaulmicola*, namely *Pulverulinacyathella* (J. Favre & Schweers ex Kuyper) Chalange & P.-A. Moreau and *Pulverulinapraticola* (Kuyper, Arnolds & P.-J. Keizer) Chalange & P.-A. Morea. These two species were transferred to *Pulverulina* by Chalange and Moreau (2023) from their previous classification under *Omphalina*. Both species can be readily distinguished from *Pulverulinaflavoalba* based on their spore size and morphology. Specifically, the spores of *Pulverulinapraticola* [(6.0-)6.5-8.0(-8.5) × (5.0-)5.5-6.5(-7.0) μm] are noticeably wider than those of *Pulverulinaflavoalba*, resulting in a significantly lower Q value (Q = 1.1-1.3, Q_mean_ = 1.2) compared to *Pulverulinaflavoalba* (Kuyper et al. 1997). Similarly, *Pulverulinacyathella* also exhibits wider spores [(5.5-)6.5-7.0 × (5.0-)6.0-6.5 μm] and are (sub)globose in shape, distinguishing them from the cylindrical spores of *Pulverulinaflavoalba* (Kuyper 1996).

### ﻿Key to 22 species belonging to nine genera of Porotheleaceae in China

**Table:** 

1	Lamellae not well developed	***Delicatulaintegrella***
–	Lamellae well developed	**2**
2	Pileocystidia present	**3**
–	Pileocystidia absent	**9**
3	Cheilocystidia not seen	***Marasmiellomycenatomentosa***
–	Cheilocystidia abundant	**4**
4	Basidiospores inamyloid *Megacollybia*	**5**
–	Basidiospores amyloid	**6**
5	Cheilocystidia digitate, narrowly or broadly clavate or sphaeropedunculate, rarely with short apical outgrowths	***Me.clitocyboidea***
–	Cheilocystidia clavate, without outgrowths	***Me.platyphylla***
6	Cheilocystidia distinctly thick-walled overall *Leucoinocybe*	**7**
–	Cheilocystidia thin-walled or slightly thick-walled in the base *Clitocybula*	**8**
7	Basidiospores narrowly ellipsoid	***L.lishuiensis***
–	Basidiospores subglobose to broadly ellipsoid	***L.subglobispora***
8	Basidiospores (5.2) 5.4–5.8–6.2 (6.5) × (4.2) 4.3–4.7–5.0 (5.1) μm, broadly ellipsoid	***C.fuscostriata***
–	Basidiospores 3.5–5.3(–5.5) × 3.5–5.0 μm, globose, subglobose to broadly elliptic	***C.familia***
9	Pileus trama sarcodimitic	**10**
–	Pileus trama not sarcodimitic	**18**
10	Basidiospores inamyloid	***Trogiavenenata***
–	Basidiospores amyloid *Gerronema***11**
11	Basidiomata distinctly small (Pileus < 9 mm in diam.)	***G.microcarpum***
–	Basidiomata moderately small (Pileus > 9 mm in diam.)	**12**
12	Pleurocystidia present	***G.chrysocarpum***
–	Pleurocystidia absent	**13**
13	Pileus blue	***G.indigoticum***
–	Pileus not blue	**14**
14	Pileus and stipe pure white	***G.albidum***
–	Pileus yellow to brown, stipe white to yellowish-brown	**15**
15	Pileus without pubescence or scales	**16**
–	Pileus densely covered with deep brown pubescence or scales	**17**
16	Cheilocystidia up to 48 μm long	***G.baishanzuense***
–	Cheilocystidia less than 35 μm long	***G.nemorale***
17	Stipe without fuscous pubescence or scales, basidiospores (6.3) 6.7–7.4–8.0 (8.5) × (3.2) 3.7–4.1–4.6 (4.8) μm	***G.zhujian***
–	Stipe with deep brown fuscous pubescence or scales, basidiospores (9.0) 9.2–10.0–11.2 (12.9) × (4.9) 5.2–5.8–6.6 (7.2) μm	***G.brunneosquamulosum***
18	Cheilocystidia absent	***Pulverulinaflavoalba***
–	Cheilocystidia present	**19**
19	Dermatocystidia inconspicuous and rare	***Pseudohydropusfloccipes***
–	Dermatocystidia abundant *Hydropus*	**20**
20	Carpophore blackening when touched or bruised	***H.nigrita***
–	Carpophore not blackening in any part when touched or bruised	**21**
21	Basidiospores ellipsoid	***H.marginellus***
–	Basidiospores broadly ellipsoid	***H.atriceps***

## ﻿Discussion

Previous molecular phylogenetic analyses of the so-called hydropoid clade and the Porotheleaceae have been conducted, based on various combinations of ITS, 28S, 18S, 5.8S, 25S, *rpb1* and *rpb2* loci (Moncalvo et al. 2002; Matheny et al. 2006, 2020; Antonín et al. 2019; Vizzini et al. 2019, 2022; Consiglio et al. 2022; Senanayake et al. 2023). In the present study, we chose three regions, namely, ITS, nrLSU and *rpb2*, to analyse phylogenetic relationships in Porotheleaceae. Phylogenetic analyses, based on a combined dataset of these three loci, indicated that *Marasmiellomycena* comprising four species and *Pulverulina*, comprising two species, constitute monophyletic clades within Porotheleaceae. We thus report new records in China for two genera, *Marasmiellomycena* and *Pulverulina*, which cover two new species and a new combination. *Marasmiellomycena* now includes two new species, namely *M.tomentosa* and *M.albodescendens*. Additionally, the species previously identified as *Porotheleumalbodescendens* has been combined as *Marasmiellomycenaalbodescendens*, representing a new combination within the *Marasmiellomycena*, all well characterised by having agaricoid basidiomata. On the basis of macromorphology and phylogenetic affinities, we have only retained one species in *Porotheleum*–the type species, *Porotheleumfimbriatum* (Pers.) Fr., which is distinguished by its fruiting clusters of small cup-shaped to tubular cream cyphelloid basidiomes that are densely crowded on a common membranous, resupinate subiculum/stroma with a broad rhizomorphic margin (Cooke 1989). Our results also agreed with Senanayake et al. (2023) that the genus *Vizzinia* contains two species *V.dominingense* and *V.nigripes*, which forms a well-supported lineage and the phylogenetic positions of *Porotheleumalbidum* and *Porotheleumparvulum* are unclear.

Morphologically, *Marasmiellomycena* is easily recognisable as an omphalinoid mushroom in the field owing to its pileus that is depressed to umbilicate at the centre, decurrent to subdecurrent lamellae, dark-coloured stipe, sarcodimitic structure and thick-walled caulocystidia with contents. *Marasmiellomycena* is most similar to *Vizzinia*, but *Vizzinia* differs in basidiomata turning brownish on handling, distinctly squamulose pileus, weakly amyloid spores and absence of cheilocystidia. *Pulverulina* resembles *Clitocybula* in being an omphalinoid basidiocarps with decurrent lamellae, but can be distinguished by pruinose stipes, inamyloid basidiospores and absence of hymenial cystidia. *Gerronema*, *Megacollybia* and *Trogia* are more similar to *Marasmiellomycena* on the basis of their sarcodimitic structure. *Marasmiellomycena* can be readily discriminated in possessing dark-coloured stipe, inamyloid basidiospores and thick-walled caulocystidia with yellow to yellowish-brown pigments. *Pulverulina* species are characterised by their inamyloid basidiospores, non-sarcodimitic structure, thin-walled caulocystidia and non-pigmented pileocystidia and caulocystidia.

Our multi-gene phylogenetic analysis divided *Gerronema* into several highly-supported clades. This finding is consistent with the analyses of Antonín et al. (2019), Vizzini et al. (2019, 2022), Matheny et al. (2020) and Na et al. (2022a), who have reported that *Gerronema* is a non-monophyletic genus comprising several unrelated clades. The type of *Gerronema* has not been sequenced so it is unclear which belongs to *Gerronema* sensu stricto. Other genera in Porotheleaceae, namely, *Chrysomycena*, *Clitocybula*, *Delicatula*, *Hydropodia*, *Hydropus*, *Leucoinocybe*, *Marasmiellomycena*, *Megacollybia*, *Pulverulina*, *Trogia* and *Vizzinia* are monophyletic in previous phylogenetic studies as well as the present one (Matheny et al. 2020; Consiglio et al. 2022; Vizzini et al. 2022; Senanayake et al. 2023). *Hydropodiasubalpina* (Höhn.) Vizzini, Consiglio & M. Marchetti, a new combination from *Hydropus*, is not related to *Hydropus* s. s.–which corresponds to the clade including the type species *Hydropusfuliginarius* (Batsch) Singer in the phylogenetic classification of Consiglio et al. (2022). In addition, Consiglio et al. (2022) consider *Hydropodia* to be sister to the *Porotheleum* clade; in our studies, however, *Hydropodia* is closer to *Pseudohydropus* and forms a sister clade.

Several species of Porotheleaceae have been reported to be edible or have toxic or ecological effects. *Megacollybiaplatyphylla* (Pers.) Kotl. & Pouzar (Dai et al. 2010), are known to be edible, whereas *Trogiavenenata* Zhu L. Yang, Yan C. Li & L.P. Tang has caused hundreds of deaths in south-western China (Yang et al. 2012). Current evidence regarding the edibility and ecological functions of other Porotheleaceae species is insufficient. Specifically, whether they engage in symbiotic or saprophytic relationships with plants, as well as their roles within ecosystems, remains unclear. Although it is uncertain if these species exhibit symbiosis (and likely absent), future studies may uncover their capabilities to promote seed germination, similar to some *Mycena* species or possessing characteristics like bioluminescence. Further research is needed to investigate the edibility and ecological role of Porotheleaceae.

## Supplementary Material

XML Treatment for Clitocybula fuscostriata

XML Treatment for Gerronema brunneosquamulosum

XML Treatment for Leucoinocybe subglobispora

XML Treatment for Marasmiellomycena albodescendens

XML Treatment for Marasmiellomycena tomentosa

XML Treatment for Pulverulina flavoalba

## ﻿Additional information

### Author ORCIDs

Qin Na https://orcid.org/0000-0001-8406-6389

Hui Zeng https://orcid.org/0000-0003-2025-844X

Yaping Hu https://orcid.org/0000-0003-1242-1139

Hui Ding https://orcid.org/0000-0003-4490-2105

Binrong Ke https://orcid.org/0009-0008-7209-7362

Zhiheng Zeng https://orcid.org/0009-0008-4208-2629

Xianhao Cheng https://orcid.org/0000-0002-5922-9913

Yupeng Ge https://orcid.org/0000-0001-5754-201X

### Data availability

All of the data that support the findings of this study are available in the main text.
